# Single-cell analysis reveals shared adaptive responses across different types of podocyte injury

**DOI:** 10.3389/fimmu.2025.1698284

**Published:** 2025-12-16

**Authors:** Liuxiao Yang, Lijun Sun, Wu Liu, Hongliang Rui, Haoran Dai, Wenbin Liu, Baoli Liu

**Affiliations:** 1 School of Traditional Chinese Medicine, Changchun University of Chinese Medicine, Changchun, China; 2 Beijing Hospital of Traditional Chinese Medicine, Capital Medical University, Beijing, China; 3 Laboratory for Clinical Medicine, Capital Medical University, Beijing, China

**Keywords:** podocyte injury, adaptive response, transcriptional program, single-cell RNA sequencing, chronic kidney disease, acute kidney injury

## Abstract

**Introduction:**

Podocytes are essential for maintaining the structural and functional integrity of the glomerular filtration barrier. Their damage constitutes a common pathological basis for proteinuria and renal function deterioration in kidney diseases. Podocyte injury exhibits marked heterogeneity in etiology, pathogenic mechanisms, and phenotypic manifestations across distinct kidney diseases, leading to different renal outcomes. However, the molecular underpinnings remain limited. Consequently, single-cell RNA sequencing (scRNA-seq) enables deconstruction of renal cell states with unprecedented resolution.

**Methods:**

Here, we integrated 16 scRNA-seq samples of human kidney tissues, totaling 73,684 cells from healthy controls and patients with IgA nephropathy (IgAN), idiopathic membranous nephropathy (IMN), and acute kidney injury (AKI). We identified 11 major cell types and analyzed podocyte injury mechanisms among these diseases, as well as their crosstalk within the glomerular niche. Key molecules were confirmed using immunohistochemistry.

**Results:**

Our analysis identified distinct podocyte injury mechanisms across diseases: HSPG2-mediated signaling from mesangial cells in IgAN, upregulation of extracellular matrix-related genes in IMN, and increased SPP1 signaling within glomeruli in AKI. Despite divergent triggers, podocytes mounted convergent adaptive responses characterized by initial structural disruption, a mitochondria-driven compensatory phase, and subsequent functional dysregulation via multiple stress pathways, culminating in irreversible damage.

**Conclusion:**

Together, our study reveals both the heterogeneous and shared adaptive responses of injured podocytes through single-cell RNA analysis, providing new insights into disease mechanisms and potential therapeutic targets.

## 1 Introduction

Podocytes (POD) are terminally differentiated, highly specialized epithelial cells in the glomerulus (1). Their primary and secondary foot processes extend from the cell body to enwrap glomerular capillaries. These processes are linked to each other through slit diaphragms, forming a distinctive and complex structure supported by stable cytoskeletons (2). Podocytes serve as critical size- and charge-selective filters within the glomerular filtration barrier, effectively preventing proteinuria (1). Constantly exposed to mechanical stress and filtered molecules, podocytes rely on complex sensing and response mechanisms to maintain homeostasis. Disruption of these regulatory pathways makes podocytes vulnerable to injury, and their limited capacity for repair and regeneration makes recovery difficult (3, 4). Early injury is marked by disorganization of the slit diaphragm and cytoskeleton, leading to foot process effacement and impaired filtration barrier function. Persistent damage causes podocyte apoptosis or detachment from the glomerular basement membrane (GBM), resulting in glomerulosclerosis and the progression of chronic kidney disease (CKD) to end-stage kidney disease (5). Additionally, podocytes communicate bidirectionally with glomerular endothelial cells, mesangial cells, and parietal epithelial cells via secreted signaling molecules, chemokines, and exosomes. Such injury disrupts this communication, causing abnormal cell function and promoting the progression of glomerular disease (6).

Numerous studies have demonstrated that podocyte injury is closely linked to the onset and progression of both CKD and acute kidney injury (AKI) (7, 8). In CKD, podocyte injury can be caused directly or indirectly by genetic factors, immune dysregulation, metabolic abnormalities, viral infections, or mechanical stress. The underlying mechanisms are complex, encompassing dysfunction of key podocyte proteins, cytoskeletal remodeling, impaired autophagy, endoplasmic reticulum stress, complement activation, accumulation of reactive oxygen species, and abnormal activation of signaling pathways—including mTOR, Wnt/β-catenin, and Notch (1, 9). Diverse manifestations following podocyte injury include foot process effacement, cell loss, hypertrophy, dedifferentiation, abnormal proliferation, epithelial-to-mesenchymal transition (EMT), detachment, apoptosis, and immune complex deposition in the subepithelial space. These pathological features differ across various CKD types (10, 11). When podocyte loss exceeds 40%, persistent proteinuria develops, which leads to glomerulosclerosis and a decline in renal function, driving CKD progression (6). In contrast, in AKI, podocyte injury is typically acute, early in onset, and often partially reversible. Common causes include ischemia-reperfusion injury, systemic infections, drug toxicity, and other toxins, which primarily mediate injury through inflammation, oxidative stress, and mitochondrial dysfunction. Typical pathological features are extensive foot process effacement and reduced expression of slit diaphragm and cytoskeletal proteins. In severe cases, podocyte apoptosis or detachment can occur, further aggravating proteinuria (12–18). Notably, animal models suggest that podocytes have significant regenerative potential after acute injury, enabling partial recovery of structure and function under favorable conditions (9). Nevertheless, if the injury persists or the pathological environment is complex, podocyte dysfunction and structural remodeling will accelerate glomerulosclerosis and fibrosis. Finally, this promotes the transition from AKI to CKD (19).

Overall, the podocyte response to injury is a complex process involving various biological mechanisms. Many of these overlap across different kidney diseases, while others are disease-specific (1). Single-cell RNA sequencing (scRNA-seq) technology has enabled researchers to study the dynamic changes and molecular characteristics of podocytes within the complex glomerular microenvironment at single-cell resolution (20). In this study, we performed a cross-disease single-cell analysis focusing on IgA nephropathy (IgAN), idiopathic membranous nephropathy (IMN), and AKI. We aim to elucidate the processes and response mechanisms underlying various types of podocyte injury, shedding new light on future research and therapies.

## 2 Methods

### 2.1 Data acquisition and quality control

Single-cell RNA sequencing data of human kidney tissues were obtained from the Gene Expression Omnibus (GEO) database (https://www.ncbi.nlm.nih.gov/geo/) and included four sample groups: healthy controls, IgA nephropathy, idiopathic membranous nephropathy, and acute kidney injury (GSE174219, GSE171314, GSE171458, and GSE174220; **Supplementary Tables S1**, **S2**) (21–24). Expression matrices from each sample were read and used to independently construct Seurat objects, followed by quality control. Cells were filtered based on the following criteria: removal of cells with fewer than 300 detected genes, exclusion of cells with UMI counts in the top 3% to avoid potential doublets or other abnormalities, retention of cells with mitochondrial gene percentages below approximately 70% (considering the typically higher mitochondrial content in renal tubular tissue), and exclusion of cells with hemoglobin gene percentages greater than 5% to reduce red blood cell contamination. The data were normalized using the LogNormalize method, and 2,000 highly variable genes were selected for scaling and principal component analysis (PCA). Dimensionality reduction was performed using uniform manifold approximation and projection (UMAP), followed by shared nearest neighbor (SNN) graph construction and clustering. All procedures were carried out using the Seurat R package (v5.1.0) (25). Doublets were identified and removed using the DoubletFinder R package (v2.0.4), completing the individual quality control workflow for each sample (26).

### 2.2 Data integration, clustering analysis, and cell annotation

All samples were merged to construct a unified Seurat object, normalized using the default LogNormalize method, and 2,000 highly variable genes were selected for scaling and PCA. Batch effects were corrected using the Harmony R package (v1.2.1) (27). An SNN graph was constructed based on the corrected data, and cell clustering was performed at a resolution of 0.7, which provided the optimal balance between capturing cell subgroups and maintaining biological relevance. Cell types were manually annotated based on known marker genes for downstream analysis (**Supplementary Table S3**) (22, 28).

### 2.3 Differential expression analysis and GO enrichment analysis

To investigate the heterogeneity of podocytes across the four groups, we used the *FindAllMarkers* function to identify marker genes. A gene was defined as a marker gene of this group if it was detected in at least 25% of cells within one group and with at least 0.25 log fold change (logFC) across groups (29), and the significance level of *P*-value < 0.05 in the Wilcoxon rank-sum test was used. Upregulated marker genes in each group were analyzed for Gene Ontology (GO) enrichment using the clusterProfiler R package (v4.12.0) (30). Significance was calculated using the hypergeometric test, and *P*-values were adjusted for multiple testing using the Benjamini–Hochberg (BH) method to control the false discovery rate (FDR). Initial filtering used pvalueCutoff = 0.05 and qvalueCutoff = 0.05, and pathways with an adjusted *P*-value < 0.05 were considered significant. The enrichment results were visualized using lollipop plots.

### 2.4 Ligand–receptor interactions of all glomerular cells

To compare cellular crosstalk among glomerular cells across the four groups, we used CellChat (v2.1.2) with the CellChatDB database to predict cell type-specific ligand–receptor interactions (31). Glomerular endothelial cells, mesangial cells, parietal epithelial cells, and podocytes were defined as both senders and receivers. Intercellular communication networks were constructed and visualized to compare ligand–receptor interactions among groups.

### 2.5 scRNA-seq analysis for IgAN

To investigate the molecular mechanisms by which mesangial cells contribute to secondary injury of podocytes in IgAN, differential expression analysis of mesangial cells was performed using the *FindMarkers* function between the IgAN and HC groups. Differentially expressed genes (DEGs) were defined as those detected in at least 25% of cells within one group, with at least 1 logFC between two groups (32), and the significance level of *P*-value < 0.05 in the Wilcoxon rank-sum test was used. Genes were ranked according to average log2 fold change values between two groups, and Gene Set Enrichment Analysis (GSEA) was conducted using the KEGG database with the *gseKEGG* function (33). Significance was defined as a *P*-value < 0.05 to assess the functional state of mesangial cells in IgAN. The *msigdbr* function was used to obtain the “KEGG CYTOKINE CYTOKINE RECEPTOR INTERACTION” gene set, and the AddModuleScore algorithm was applied to calculate module scores for each cell. The scores were compared across the four groups and visualized using a bubble plot. Cell communication analysis was performed using the same approach as mentioned above, focusing on interactions with glomerular endothelial cells, mesangial cells, and parietal epithelial cells as signal sources and podocytes as the target. *HSPG2* expression differences in mesangial cells across the four groups were visualized using a dot plot. Expression levels were validated using the Wilcoxon rank-sum test with the “Reich IgAN Glom” dataset (probe ID: 201655_s_at) from the Nephroseq v5 platform (https://www.nephroseq.org/resource/login.html). Pearson correlation analysis was performed with the “Ju CKD Glom” dataset to evaluate the association between glomerular *HSPG2* expression and serum creatinine in patients with IgAN.

### 2.6 scRNA-seq analysis for IMN

To investigate the molecular characteristics of podocytes in IMN, we applied the differential expression analysis approach used for mesangial cells in IgAN to identify DEGs between podocytes in the IMN and HC groups. GO enrichment analysis was performed based on the upregulated DEGs (same method as before). The *msigdbr* function was used to obtain the “REACTOME EXTRACELLULAR MATRIX ORGANIZATION” gene set, and the AddModuleScore algorithm was applied to calculate module scores for each cell. The scores were compared across the four groups and visualized using a violin plot. Dot plots were used to show the expression differences of extracellular matrix (ECM)-related genes within podocytes across the four groups, and box plots were generated to further visualize significantly altered collagen genes. Expression differences of *COL3A1*, *COL4A3*, and *COL4A4* between IMN and HC groups were validated using the Nephroseq v5 platform “Ju CKD Glom” dataset and assessed with the Wilcoxon rank-sum test.

### 2.7 scRNA-seq analysis for AKI

Cellular crosstalk among glomerular endothelial cells, mesangial cells, parietal epithelial cells, and podocytes was analyzed using the same method described earlier, focusing on ligand–receptor pairs enhanced in AKI. *SPP1* expression differences across glomerular cell types (glomerular endothelial cells, mesangial cells, parietal epithelial cells, and podocytes) as well as renal tubular epithelial cells were visualized using violin, UMAP, and heatmap plots. Pearson correlation analysis using the Nephroseq v5 platform “Sampson Nephrotic Syndrome Glom” dataset was conducted to examine the association between glomerular *SPP1* expression and glomerular filtration rate (GFR) in patients with nephrotic syndrome.

### 2.8 Differential expression analysis and trajectory analysis of POD subpopulations

To investigate the heterogeneity among POD subpopulations, marker genes were identified using the *FindAllMarkers* function. A gene was the subpopulation marker if it was detected in at least 25% of one subcluster and with at least 0.25 logFC between two subpopulations (29), and the significance level of the BH-adjusted *P*-value < 0.05 in the Wilcoxon rank-sum test was used. GO enrichment analysis was performed on the upregulated marker genes in each POD subpopulation (same method as before). Hallmark gene sets were downloaded using the *msigdbr* function. Enrichment scores were calculated using the GSVA R package (v1.48.0), and a heatmap was generated for visualization. Using custom stress-related and ferroptosis-related gene sets (**Supplementary Data S1**) (34–40), gene set scores for individual cells were computed with the AddModuleScore algorithm. To assess the state transitions of podocytes under different injury conditions, CytoTRACE (v0.3.3) was applied (41), which identified POD1 as the trajectory starting point. Monocle 3 (v1.3.1) was used to construct cell trajectories and validated with other trajectory inference tools (42), including Monocle2 (v2.34.0) (43), SCORPIUS (v1.0.9) (44), and Vector (R script from GitHub, https://github.com/jumphone/Vector) (45). Trajectory-associated genes were selected based on *q*-value < 0.05 and Moran’s I > 0.25, and their dynamic expression was visualized using a density plot and heatmap.

### 2.9 Patients

Kidney biopsies were collected from patients with IgAN, IMN, and AKI at Beijing Hospital of Traditional Chinese Medicine. Formalin-fixed, paraffin-embedded IgAN and AKI biopsies were subjected to immunohistochemical analyses, and glutaraldehyde-fixed, resin-embedded IMN biopsies were examined by transmission electron microscopy. Glomeruli from healthy controls served as comparators. This study was conducted in accordance with the Declaration of Helsinki and was approved by the Ethics Committee of Beijing Hospital of Traditional Chinese Medicine (number: 2024BL02-043).

### 2.10 Electron microscopy

Kidney tissues were fixed in 2.5% glutaraldehyde at 4 °C for 24 hours, followed by post-fixation with 1% osmium tetroxide. The samples were then dehydrated and embedded in epoxy resin. Ultrathin sections (~70 nm) were stained with uranyl acetate and lead citrate, and observed and imaged using a JEM-1400 transmission electron microscope (JEOL, Japan).

### 2.11 Immunohistochemistry

Human kidney tissue samples were fixed in 4% paraformaldehyde, embedded in paraffin, and sectioned at 4 μm thickness. Sections were deparaffinized in xylene, rehydrated through a graded ethanol series, and subjected to heat-induced antigen retrieval in citrate buffer (pH 6.0). Endogenous peroxidase activity was quenched with 3% hydrogen peroxide, followed by blocking of nonspecific binding with 5% bovine serum albumin (BSA). Primary antibodies (anti-HSPG2, 1:100, Thermo Fisher Scientific, USA; anti-SPP1, 1:1000, Thermo Fisher Scientific, USA) were applied and incubated overnight at 4 °C. The following day, sections were incubated with HRP-conjugated secondary antibodies at room temperature for 30 minutes, followed by DAB development and hematoxylin counterstaining. Sections were dehydrated, mounted, and imaged using a light microscope. Patient and antibody information is listed in **Supplementary Tables S4** and **S5**.

### 2.12 Statistical analysis

Comparisons between two groups were performed using the Mann-Whitney U test, and correlations between variables were assessed using Pearson analysis. *P* < 0.05 was considered statistically significant.

## 3 Results

### 3.1 Classification and identification of kidney and glomerular cell subpopulations

**Figure 1:**
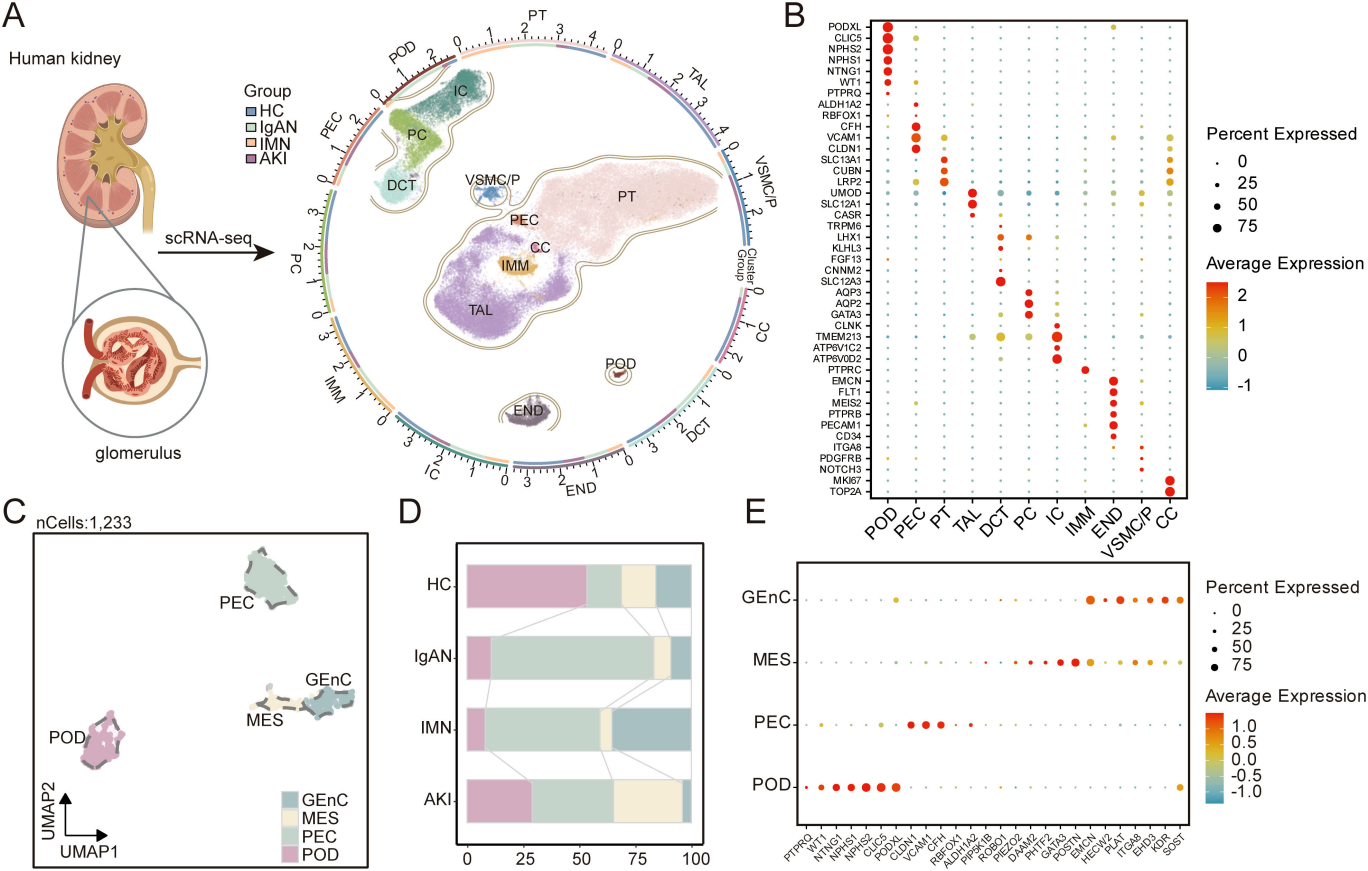
Identification of kidney and glomerular cell types. **(A)** UMAP plot visualizing kidney cells identified by clustering. Cells were annotated based on marker gene expression, including POD, PEC, PT, TAL, DCT, PC, IC, IMM, END, VSMC/P, and CC. **(B)** Dot plot showing marker gene expression within each kidney cell type. Dot size indicates the proportion of expressing cells, and color intensity reflects average expression levels. **(C)** UMAP plot of glomerular cells annotated as GEnC, MES, PEC, and POD based on marker gene expression. **(D)** Stacked bar charts representing proportions of glomerular cells in each group. **(E)** Dot plot showing marker gene expression within each glomerular cell type. UMAP, Uniform manifold approximation and projection; POD, podocytes; PEC, parietal epithelial cells; PT, proximal tubule cells; TAL, thick ascending limb cells; DCT, distal convoluted tubule cells; PC, principal cells; IC, intercalated cells; IMM, immune cells; END, endothelial cells; VSMC/P, vascular smooth muscle cells and pericytes; CC, cycling cells. Parts of panel **(A)** were created with BioRender.com.

We collected 16 scRNA-seq samples, including HC (n = 4), IgAN (n = 4), IMN (n = 6), and AKI (n = 2). Clinical information for these samples is provided in **Supplementary Table S1**. After stringent quality control, we retained a total of 73,684 high-quality single cells for analysis. Through unsupervised clustering combined with marker gene annotation, we identified 11 major cell populations: podocytes (POD), parietal epithelial cells (PEC), proximal tubule cells (PT), thick ascending limb cells (TAL), distal convoluted tubule cells (DCT), principal cells (PC), intercalated cells (IC), immune cells (IMM), endothelial cells (END), vascular smooth muscle cells and pericytes (VSMC/P), and cycling cells (CC) (**Figures 1A, B**). Focusing further on the glomerular region, we identified four resident glomerular cells based on classical marker genes: glomerular endothelial cells (GEnC), mesangial cells (MES), PEC, and POD (**Figures 1C–E**).

### 3.2 Heterogeneity of podocyte injury accompanied by changes in VEGFA secretion

**Figure 2:**
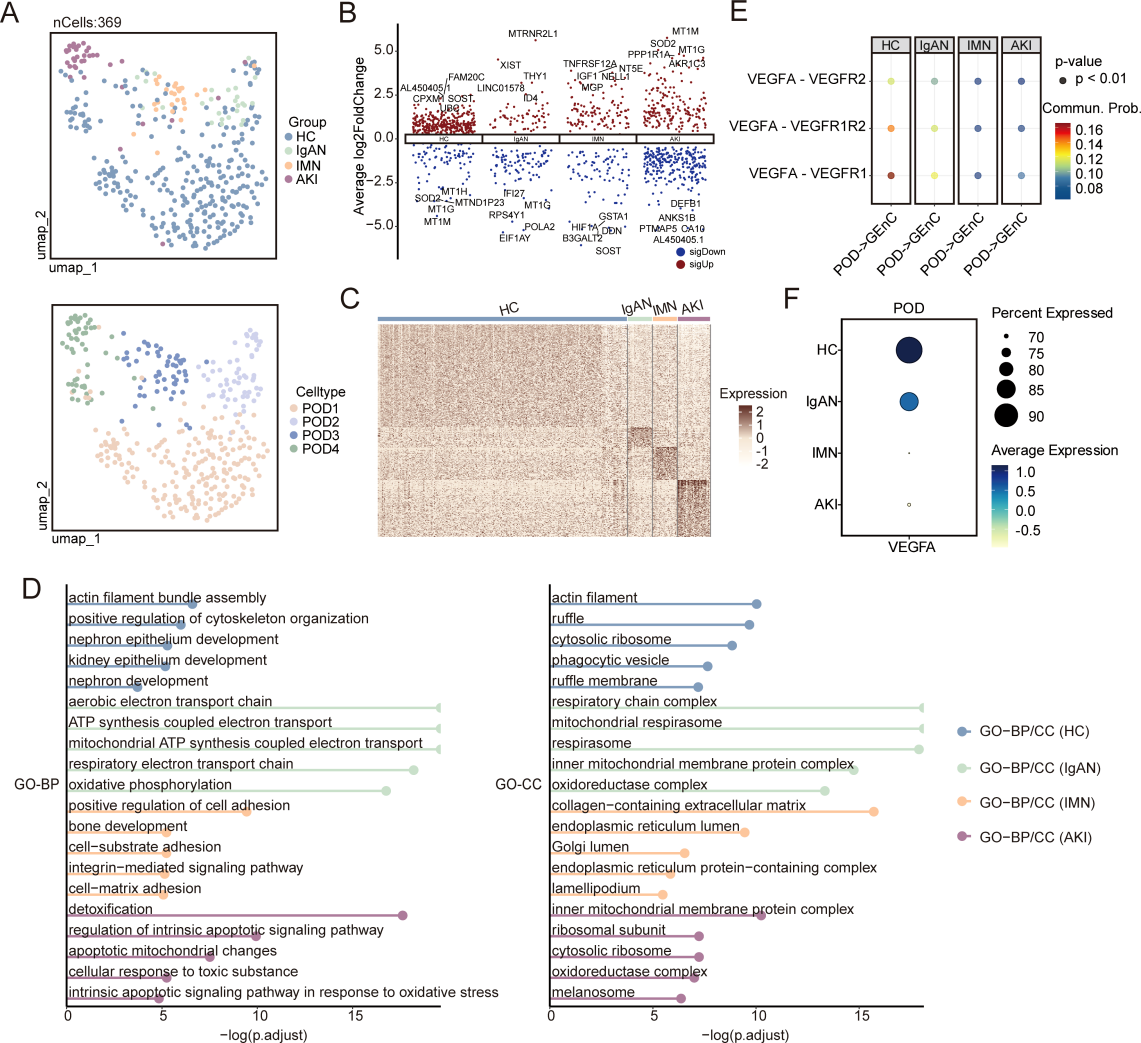
Identification of POD subpopulations and analysis of differential expression and GO enrichment. **(A)** UMAP plot of 369 podocytes, with the upper panel clustered by four sample types (HC, IgAN, IMN, and AKI) and the lower panel clustered as POD1 (205 cells), POD2 (58 cells), POD3 (54 cells), and POD4 (52 cells). **(B)** Volcano plots presenting DEGs within podocytes in each group, highlighting key upregulated and downregulated genes. **(C)** Heatmap showing upregulated DEGs across groups. **(D)** Bar plots showing −log10 (adjusted *P*-values) of selected GO pathway enrichments in each group. **(E)** Comparison of selected ligand**–**receptor interactions from podocytes to glomerular endothelial cells across groups. **(F)** Dot plot showing *VEGFA* expression levels in podocytes across groups. GO, Gene Ontology; HC, healthy control; IgAN, IgA nephropathy; IMN, idiopathic membranous nephropathy; AKI, acute kidney injury; DEGs, differentially expressed genes.

To study the characteristics of different types of podocyte injury, we identified four subpopulations (POD1–POD4) from 369 podocytes and classified these cells into four groups based on sample type: HC, IgAN, IMN, and AKI (**Figure 2A**). We performed differential expression analysis to identify genes specific to each group (**Figure 2B**; **Supplementary Data S2**). GO enrichment analysis of these upregulated genes showed that the HC group was mainly associated with nephron development. In contrast, the IgAN group was enriched in energy metabolism pathways, such as the electron transport chain and ATP synthesis. The IMN group showed increased activity in ECM synthesis and secretion, while the AKI group was marked by activation of stress-related pathways, including oxidative stress, detoxification metabolism, and apoptosis (**Figures 2C, D**). These results highlight the heterogeneity of podocyte injury across different kidney diseases. Furthermore, CellChat analysis revealed that VEGFA signaling from podocytes to glomerular endothelial cells progressively declines as podocyte injury worsens (**Figure 2E**). Consistently, *VEGFA* expression within podocytes decreased accordingly (**Figure 2F**). These results suggest that reduced VEGFA secretion by podocytes may impair the paracrine regulation of glomerular endothelial cells, thereby contributing to glomerular filtration barrier injury (1).

### 3.3 HSPG2 secreted from mesangial cells mediates mild podocyte injury in IgAN

**Figure 3:**
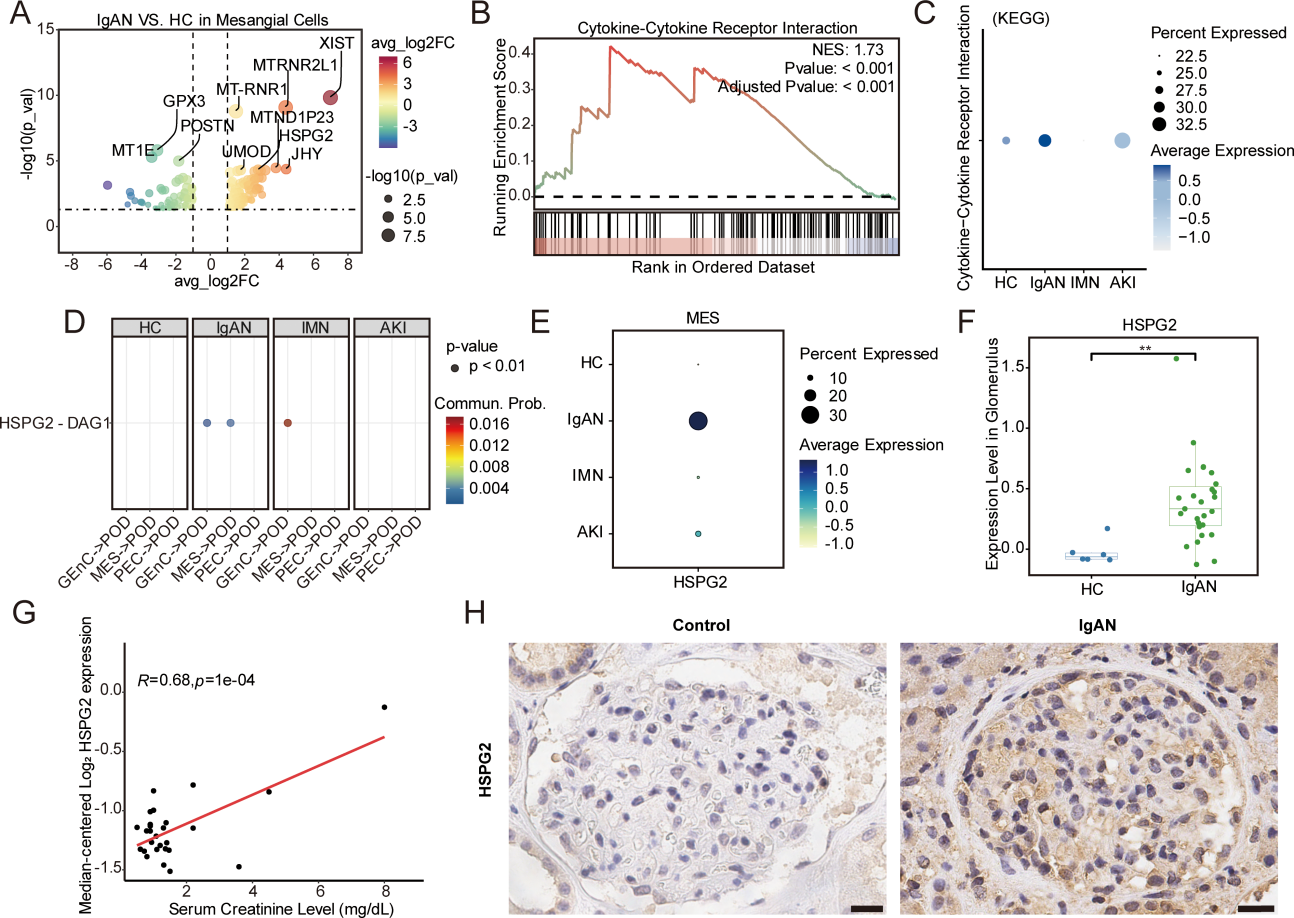
Differential expression analysis in mesangial cells and validation of HSPG2 in IgAN. **(A)** Volcano plot presenting the top 10 significantly changed genes within mesangial cells from IgAN patients. Vertical lines indicate thresholds at average log2 fold change values of > 1 and < −1, while the horizontal line represents a *p*-value cutoff of 0.05. Point color indicates the direction and magnitude of expression changes, and point size reflects significance. **(B)** GSEA of mesangial cells in IgAN compared to HC. **(C)** Dot plot showing activity scores of specific KEGG pathway gene sets in mesangial cell subpopulations, grouped by sample types. **(D)** Comparison of selected ligand**–**receptor pairs from glomerular endothelial cells, mesangial cells, and parietal epithelial cells targeting podocytes across groups. **(E)** Dot plot showing *HSPG2* expression within mesangial cells across groups. **(F)***HSPG2* expression is significantly higher in glomeruli of IgAN patients compared to HC (*P* = 0.00114). **(G)** Correlation analysis between glomerular *HSPG2* expression and serum creatinine levels in IgAN patients. **(H)** Immunohistochemistry showing positive HSPG2 staining in the glomerular mesangial matrix of IgAN kidneys, compared with minimal staining in kidneys from healthy controls; Scale bars, 20 μm. GSEA, Gene Set Enrichment Analysis; KEGG, Kyoto Encyclopedia of Genes and Genomes. *P < 0.05, **P < 0.01.

To elucidate the molecular characteristics of injured mesangial cells in IgAN, we compared gene expression in mesangial cells from the IgAN and HC groups (**Figure 3A**; **Supplementary Data S3**). GSEA revealed significant activation of the cytokine–cytokine receptor interaction pathway in the IgAN group (**Figures 3B, C**), potentially affecting neighboring cells. Moreover, CellChat analysis identified HSPG2-DAG1 as a ligand–receptor pair between mesangial cells and podocytes, specifically in the IgAN group (**Figure 3D**). Mesangial cells in IgAN also showed the highest expression of *HSPG2* among all groups (**Figure 3E**), which was further supported by Nephroseq database analysis, demonstrating significantly elevated *HSPG2* mRNA levels in the glomeruli of IgAN patients compared to HC (**Figure 3F**). These elevated *HSPG2* levels positively correlated with serum creatinine (**Figure 3G**). Immunohistochemistry further revealed stronger HSPG2 staining in the glomerular mesangial and extracellular matrix regions of IgAN kidneys, suggesting that HSPG2 can be produced by mesangial cells and secreted into the mesangial matrix (**Figure 3H**). Collectively, these results suggest that mesangial cell-secreted HSPG2 is associated with mild podocyte injury and declining kidney function in IgAN.

### 3.4 Aggravated podocyte injury with increased ECM synthesis and secretion in IMN

**Figure 4:**
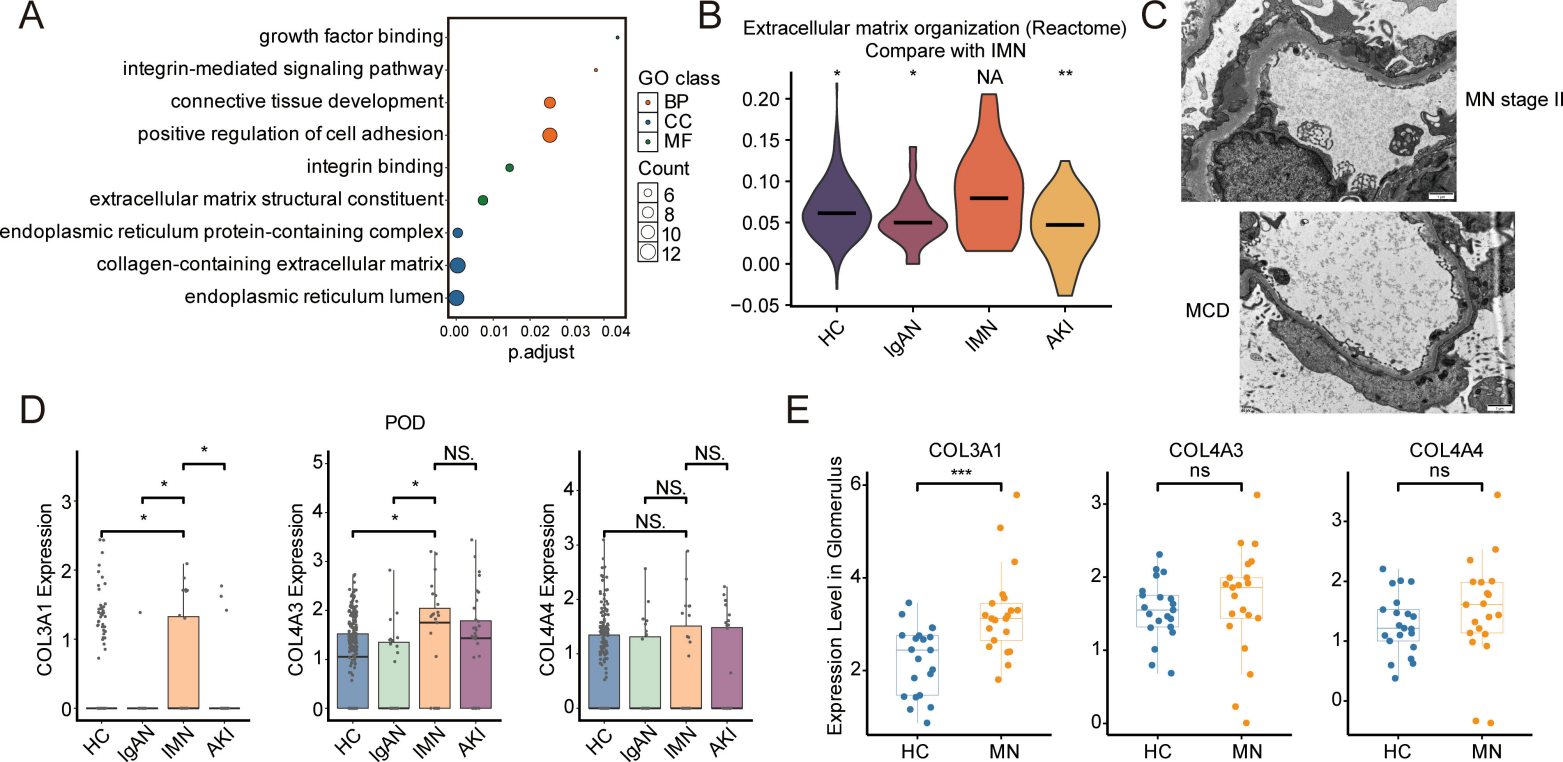
Molecular features of podocytes and ultrastructural changes of the GBM in IMN. **(A)** GO enrichment analysis of upregulated DEGs within podocytes from IMN compared to HC. **(B)** Violin plots displaying podocyte module scores for specific Reactome pathways across groups. **(C)** Transmission electron microscopy images displaying GBM thickening in IMN patients, in contrast to MCD; scale bars: 1 μm. **(D)** Boxplots of *COL3A1*, *COL4A3*, and *COL4A4* expression within podocytes across groups. **(E)***COL3A1* expression is significantly elevated in IMN glomeruli compared to HC (*P* = 0.000593), while *COL4A3* and *COL4A4* show no significant differences. GBM, glomerular basement membrane; MCD, minimal change disease. *P < 0.05, **P < 0.01, ***P < 0.001.

To elucidate the molecular characteristics of injured podocytes in IMN, we performed differential expression analysis between the IMN and HC groups, followed by GO enrichment analysis of upregulated DEGs. The results showed significant enrichment of pathways related to ECM synthesis and secretion in the IMN group, with upregulation of collagen- and integrin-related pathways (**Figures 4A, B**; **Supplementary Data S4**). These molecular changes correspond well with the characteristic GBM thickening observed under electron microscopy (**Figure 4C**). Notably, collagen genes *COL3A1* and *COL4A3* were significantly upregulated in the IMN group (**Figure 4D**), while other ECM-related genes also tended to be upregulated (**Supplementary Figure S1**). Analysis of the Nephroseq database confirmed significantly higher *COL3A1* expression in IMN glomeruli compared to HC; *COL4A3* expression was also increased, though not significantly (**Figure 4E**). These findings suggest that increased ECM synthesis and secretion contribute to GBM thickening in IMN.

### 3.5 SPP1-induced changes in the glomerular microenvironment cause severe podocyte injury in AKI

**Figure 5:**
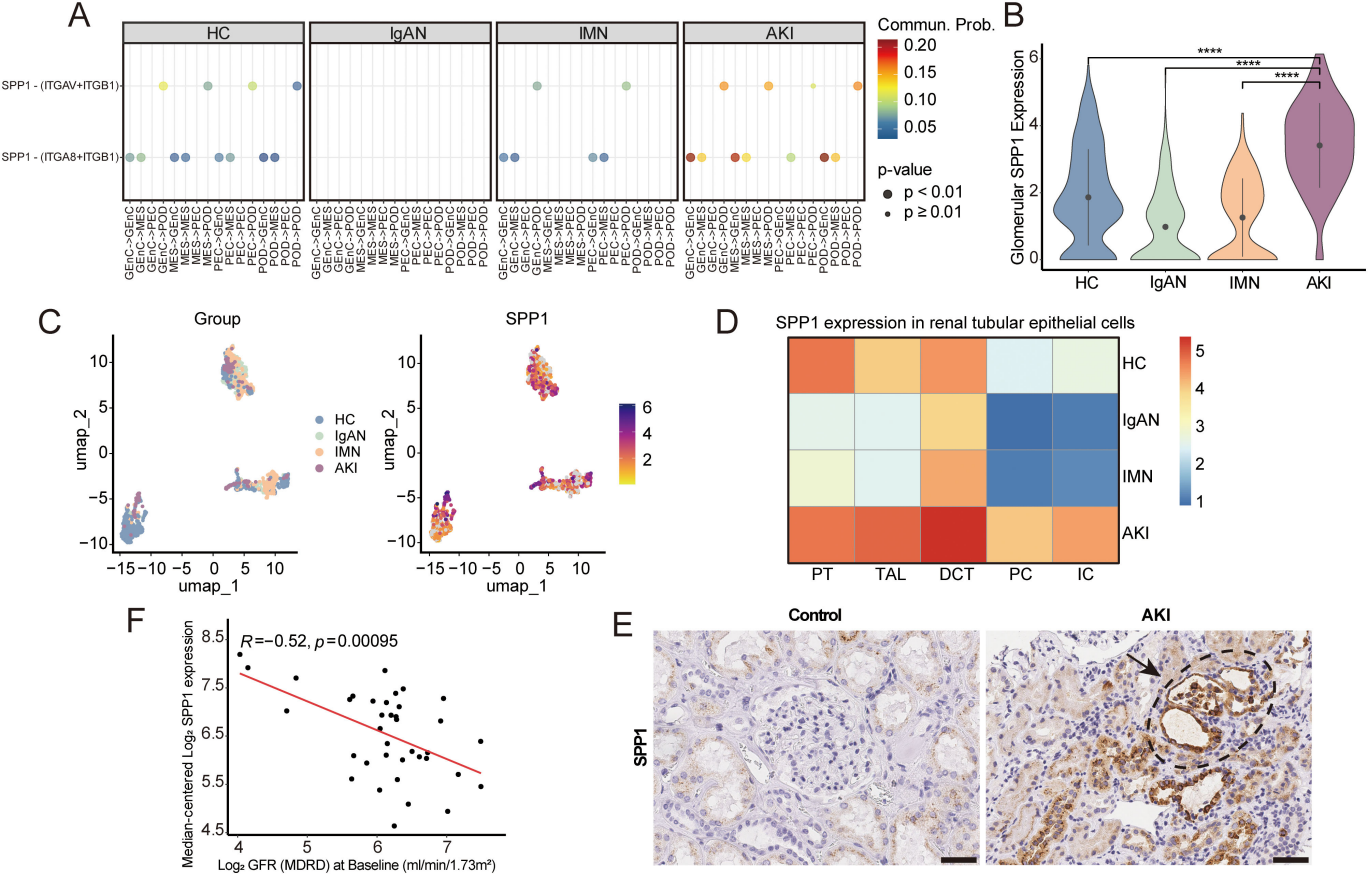
Expression and correlation analysis of SPP1 in AKI glomeruli. **(A)** Comparison of selected ligand**–**receptor pairs among glomerular cell types, analyzed as both senders and receivers across groups. **(B)** Violin plots displaying *SPP1* expression levels within glomerular cells across groups. **(C)** UMAP plots visualizing *SPP1* expression within glomerular cells across groups. **(D)** Heatmap showing *SPP1* expression in tubular epithelial cells across groups. **(E)** Immunohistochemistry showing positive SPP1 staining in the glomeruli and adjacent tubules of AKI kidneys, compared with minimal staining in kidneys from healthy controls; Scale bars, 50 μm. **(F)** Correlation between glomerular *SPP1* expression and GFR in patients with nephrotic syndrome. GFR, glomerular filtration rate. ****P < 0.0001.

To study the changes of the glomerular microenvironment in AKI, we performed CellChat analysis. The results revealed significant activation of SPP1 signaling in AKI glomeruli (**Figure 5A**), with the highest expression within podocytes and elevated levels within other glomerular cells (**Figures 5B, C**). The complete CellChat results for all previously described glomerular cells in this study are provided in **Supplementary Figure S2**. Moreover, we found that *SPP1* expression was also elevated in tubular epithelial cells (**Figure 5D**). Immunohistochemistry further confirmed enhanced SPP1 staining in both glomeruli and adjacent tubules of AKI kidneys, with higher expression than in healthy controls (**Figure 5E**). Furthermore, analysis of the Nephroseq database showed a negative correlation between *SPP1* expression and GFR (**Figure 5F**). Together, these findings indicate that SPP1 expression is elevated in both glomerular and tubular cells in AKI, and may contribute to accelerating renal function decline.

### 3.6 Trajectory and adaptive responses following podocyte injury

**Figure 6:**
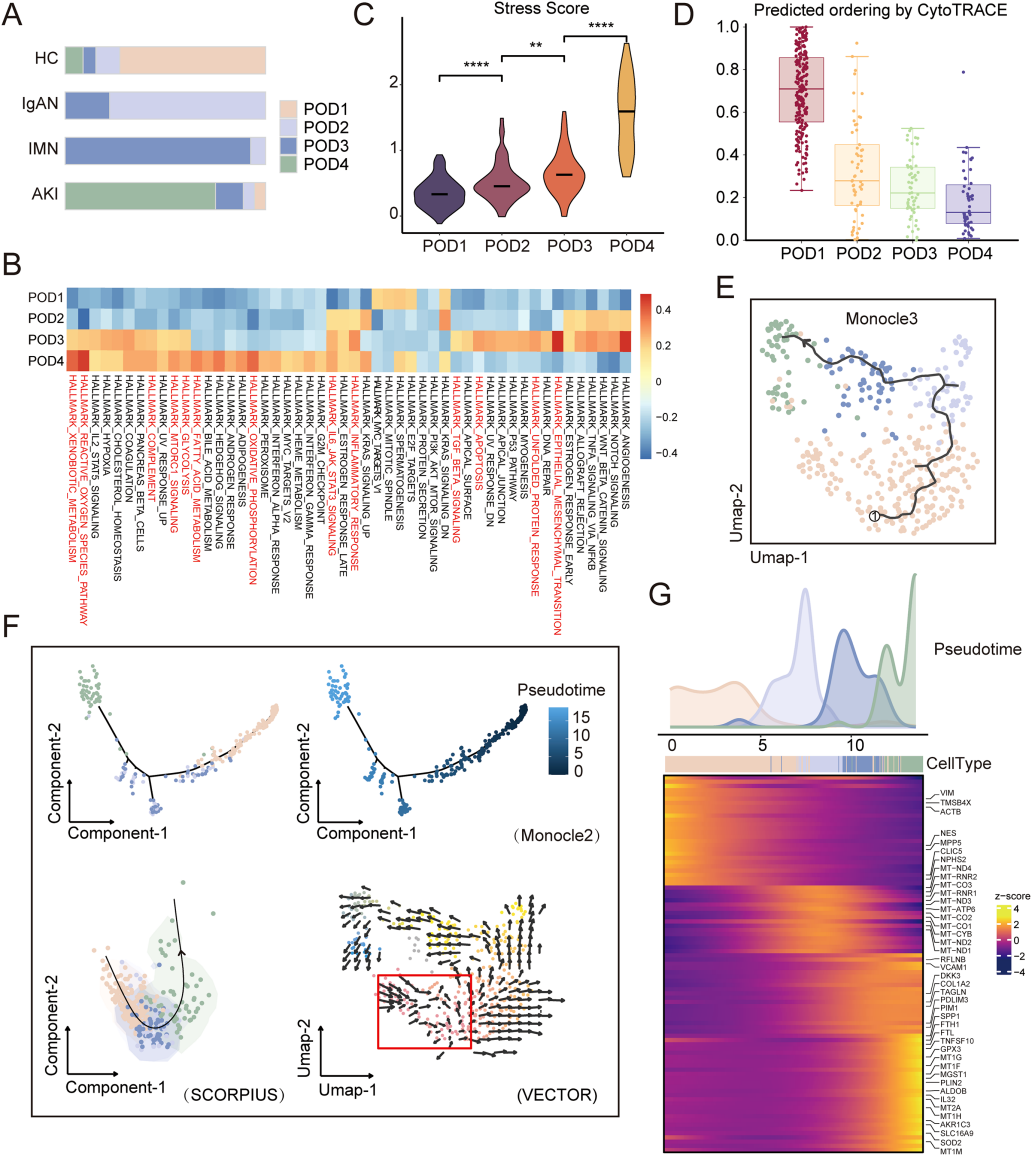
Distribution, functional enrichment, and trajectory analysis of podocyte subpopulations. **(A)** Stacked bar plots representing proportions of POD subpopulations across groups. **(B)** Heatmap showing pathway enrichment in POD subpopulations based on Hallmark gene sets. The color gradient from blue to red indicates increasing pathway activity. **(C)** Violin plots displaying stress-related gene module scores across POD subpopulations. **(D)** Distribution of CytoTRACE scores across POD subpopulations. **(E)** Monocle 3 illustrates the trajectory of podocytes from healthy to disease conditions, with cells colored by subpopulation. **(F)** Pseudotime trajectory plots of podocytes generated with Monocle 2, SCORPIUS, and VECTOR algorithms (the red box marks the starting point). **(G)** Cell-type distribution along the pseudotime trajectory, with a heatmap showing dynamic gene expression changes.

Our study identified four podocyte subpopulations (POD1, POD2, POD3, and POD4), which mainly consisted of cells from the HC, IgAN, IMN, and AKI groups, respectively (**Figure 6A**). We performed differential expression analysis for each subpopulation, followed by GO enrichment analysis on the upregulated genes (**Supplementary Figure S3A**; **Supplementary Data S5**). HALLMARK gene set scoring showed low pathway activity in POD1 and POD2, suggesting mild podocyte injury. In POD3, pathways related to inflammation, apoptosis, and EMT were highly activated, while POD4 was characterized by metabolic dysfunction and oxidative stress, indicating progressively severe injury (**Figure 6B**). Additionally, custom gene scores related to stress and ferroptosis progressively increased from POD1 to POD4, supporting these findings (**Figure 6C**; **Supplementary Figure S3B**; **Supplementary Data S1**). Furthermore, we also focused on PECs and observed the presence of progenitor-like podocytes within them, suggesting that these PECs may play a role in podocyte repair following injury (**Supplementary Figure S4**).

**Figure 7:**
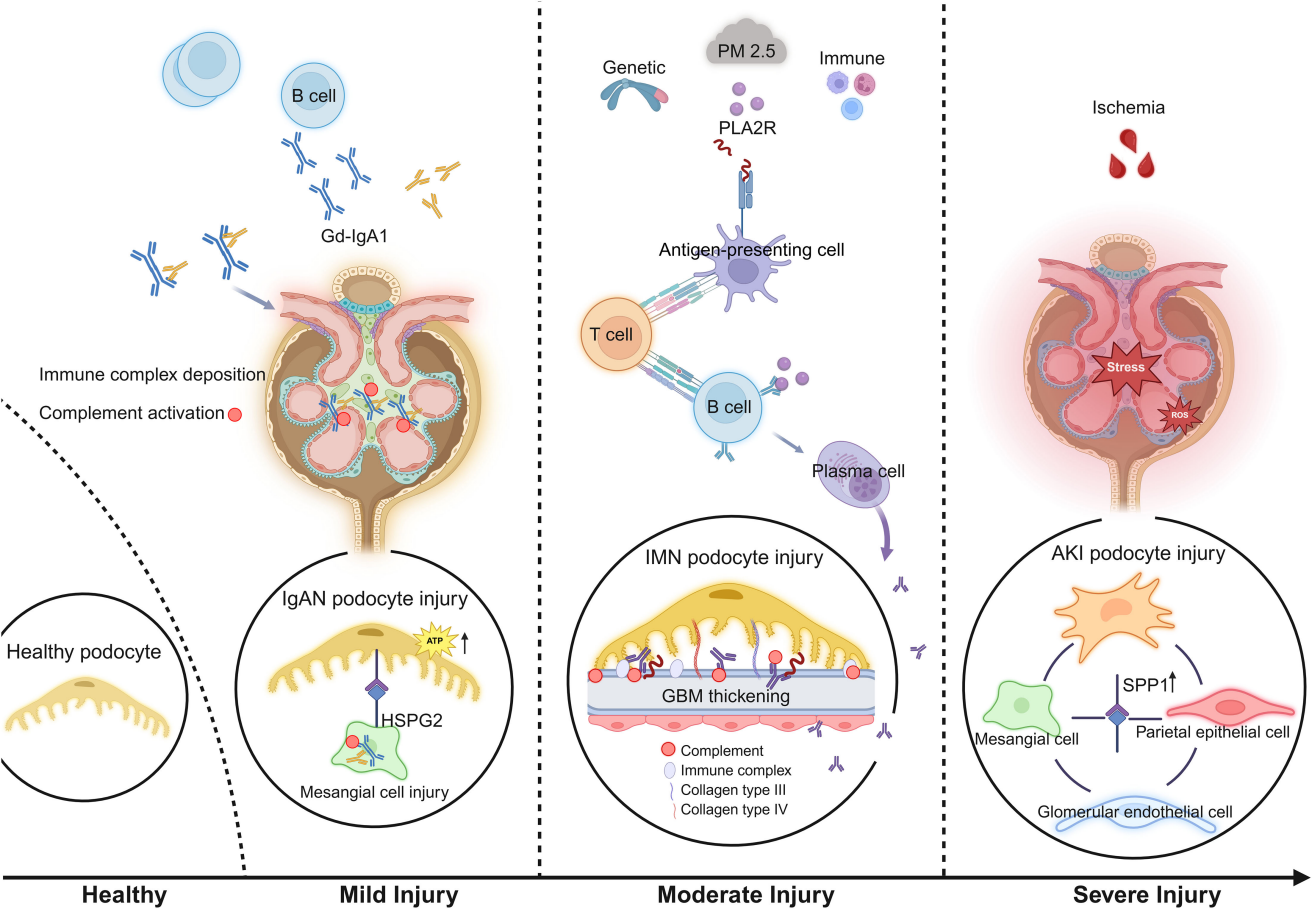
A schematic illustrating podocyte injury features and molecular pathways in three diseases. In IgAN, mild podocyte injury mediated by HSPG2 occurs secondary to mesangial cell damage. In IMN, podocytes exhibit moderate injury with increased ECM synthesis and secretion. In AKI, podocytes experience acute severe injury with significant activation of SPP1 signaling in the glomerulus. This figure highlights the heterogeneity of podocyte injury across these diseases and points to potential therapeutic targets. ECM, extracellular matrix. The figure was created with BioRender.com.

Next, CytoTRACE analysis suggested that POD1 represents the differentiation starting point, with cells gradually transitioning toward POD4 (**Figure 6D**), indicating progressively worsening injury rather than stemness changes. Trajectory analysis using Monocle3 illustrated the differentiation trajectory of the four subpopulations (**Figure 6E**), with consistent results confirmed by Monocle2, SCORPIUS, and VECTOR algorithms (**Figure 6F**). The results showed that podocytes followed a continuous trajectory, suggesting similar adaptive responses to these injuries. However, the differentiation trajectories were distinct among the three diseases (**Supplementary Figures S3C–F**). A total of 89 genes were significantly associated with pseudotime (**Figure 6G**). During the early stage of the trajectory (POD1 to POD2), genes involved in maintaining podocyte cytoskeletal stability and slit diaphragm integrity (e.g., *NPHS2*, *CLIC5*) were downregulated (46–52), suggesting the onset of podocyte structural and functional impairment. In the mid-stage (POD2), several mitochondrial genes (e.g., *MT-CYB*, *MT-ND4*) were upregulated (53), indicating activation of mitochondrial compensatory mechanisms to sustain energy balance and cellular function following initial damage. At latter stages (POD3 and POD4), podocytes exhibited a multidimensional adaptive response, characterized by the significant upregulation of genes involved in antioxidant defense (*SOD2*, *GPX3*, *MT1/2*) (54, 55), cytoskeletal remodeling (*TAGLN*, *PDLIM3*, *RFLNB*) (56–58), metabolic regulation (*ALDOB*, *SLC16A9*, *PIM1*, *PLIN2*, *AKR1C3*) (59–63), inflammation (*IL32*, *VCAM1*, *TNFSF10*) (64–66), and fibrosis (*COL1A2*, *DKK3*, *SPP1*) (67–69). These changes suggest that under persistent injury, podocytes attempt to maintain functional stability through multiple adaptive mechanisms but ultimately progress toward irreversible damage. Finally, we present a schematic diagram summarizing podocyte injury features and potential mechanisms among the three diseases (**Figure 7**).

## 4 Discussion

This study performed an initial analysis of injury mechanisms in podocytes for HC, IgAN, IMN, and AKI, revealing different features and their resulting similar responses under different pathological conditions. In IgAN, we observed mesangial cell activation, with HSPG2 secreted by mesangial cells and deposited in the mesangial matrix, leading to secondary podocyte injury. The Heparan Sulfate Proteoglycan 2 (*HSPG2*) gene encodes Perlecan, a heparan sulfate proteoglycan primarily localized in the mesangial matrix and Bowman’s capsule, playing an important role in ECM maturation and stability. Using CellChat analysis, we found that the HSPG2-DAG1 axis is specifically present between mesangial cells and podocytes, suggesting that this axis may play a role in podocyte injury in IgAN. Therefore, we hypothesize that the upregulation of HSPG2 may interact with DAG1 (Dystroglycan 1) on podocytes, leading to podocyte activation. DAG1 is responsible for linking the cell membrane to the ECM, helping podocytes adhere to the ECM. The stability of this connection is critical for maintaining the integrity of the podocyte cytoskeleton. Overactivation of DAG1 may disrupt this connection, compromising the integrity of the podocyte cytoskeleton and leading to cytoskeletal rearrangement. This mechanism may help explain clinical observations reported in the original literature, where IgAN patients typically present with mild to moderate proteinuria (0.27–2.57 g/24 h) (22, 70–72). Although our current study can only show an association between HSPG2 and podocyte injury, making it difficult to establish causality, the existing mechanisms suggest that HSPG2 may indirectly contribute to podocyte injury by modulating the connection between podocytes and the ECM.

Podocyte damage in IMN patients worsened, resulting in a marked increase in proteinuria (1.18–11.35 g/24 h) (23). Injured podocytes upregulate several ECM-related genes, including *COL3A1* and *COL4A3*, which encode type III and IV collagen, respectively (73, 74). This suggests that podocytes contribute to ECM synthesis and remodeling, supporting the repair of the glomerular filtration barrier. Under normal conditions, podocytes both produce ECM proteins and rely on them for structural support and signaling, interactions essential for maintaining barrier stability (75–79). Following injury, excessive accumulation of ECM components such as collagen and glycoproteins may occur. These deposits can encapsulate immune complexes, reducing direct damage to neighboring cells. However, excessive ECM deposition promotes glomerular fibrosis, thereby accelerating disease progression (80). ECM communicates bidirectionally with the podocyte cytoskeleton through integrin-mediated adhesion structures, thereby influencing the dynamics of the podocyte cytoskeleton (81). This suggests that the upregulated ECM may trigger cytoskeletal rearrangement in podocytes via integrin-related signaling pathways, such as the Focal Adhesion Kinase (FAK) pathway and Rho GTPase signaling pathways (82).

In this study, AKI was induced by ischemia (24), with podocyte damage being the most severe among the three diseases. However, patients typically presented with normal or trace proteinuria, likely because AKI primarily affected the renal tubules. Rapid normalization of serum creatinine following treatment suggested that renal dysfunction was reversible and that the lesions were still at an early stage. Although podocytes sustained damage, their regenerative capacity after acute injury helped maintain the integrity of the filtration barrier, resulting in only trace proteinuria. We observed significant upregulation of SPP1 in renal tubular epithelial cells, as well as in glomeruli, particularly within podocytes, highlighting its critical role in AKI-associated renal injury. Osteopontin (OPN), encoded by the secreted phosphoprotein 1 (*SPP1*) gene, functions both as an ECM component and a soluble pro-inflammatory cytokine (83). Packed with mitochondria and relying on oxidative phosphorylation, proximal tubular epithelial cells are particularly vulnerable to ischemic and hypoxic injury, making them the first site of damage in AKI (84). Injured tubular cells may secrete SPP1 as a soluble inflammatory mediator, which could potentially feed back to the glomerulus, further promoting or exacerbating glomerular and podocyte injury (85). On one hand, SPP1 may recruit immune cells, such as macrophages and T cells, by binding to receptors on these immune cells (e.g., CD44 or T-cell receptors), promoting the release of cytokines such as TNF-α, IL-6, and IL-1β, which further exacerbates the local inflammatory response and contributes to podocyte injury (86). On the other hand, SPP1 is also highly expressed in glomerular endothelial cells, mesangial cells, and parietal epithelial cells, where it binds to the ITGAV-ITGB1 integrin receptor on podocytes, activating integrin signaling pathways that trigger cytoskeletal rearrangement. When the ITGAV-ITGB1 αvβ1 integrin complex is activated, it triggers the integrin signaling pathway, leading to cytoskeletal rearrangement, for example, by activating FAK and Rho GTPases, promoting actin polymerization, altering podocyte morphology and adhesiveness, and consequently affecting their function and migration (77, 87). Upregulation of *SPP1* expression was further confirmed in a mouse renal ischemia-reperfusion model, and this protein is closely associated with renal fibrosis (69, 88). In conclusion, SPP1 not only reflects acute stress but may also drive the progression of AKI to CKD. SPP1, as a damage-associated molecule, is elevated in AKI and other renal diseases, and has also been observed in injuries to other organs (89–92). Being a soluble secreted protein that can be excreted in urine, it may serve as an indicator of kidney damage, particularly in the early stages of AKI. As such, it holds promise as both a biomarker and a therapeutic target for evaluating AKI-associated renal injury and its prognosis.

Nevertheless, we found that injured podocytes exhibit common features. First, we observed that increasing podocyte damage was accompanied by a gradual decrease in VEGFA secretion. This reduction weakened the maintenance of glomerular endothelial fenestrations, disrupted the filtration barrier, and ultimately aggravated proteinuria (1). These findings suggest that podocyte injury exacerbates glomerular damage through disrupted podocyte–endothelial signaling. Therefore, stabilizing podocyte homeostasis or reestablishing podocyte–endothelial signaling may help preserve the filtration barrier and slow proteinuria progression. A reduction in VEGFA may serve as an early marker of podocyte injury, reflecting impaired renal filtration function, and its levels may also correlate with glomerular damage and disease progression. Monitoring VEGFA levels can assist in the early diagnosis and assessment of kidney injury severity. For instance, in patients with lupus nephritis, VEGF expression in the kidneys is considered a marker of renal injury and can predict the risk of short-term renal function loss (93), further supporting VEGFA’s potential as a biomarker. Second, pseudotime analysis showed a continuous trajectory of podocytes from HC, IgAN, IMN, and AKI in reduced-dimensional space. The trajectory progresses from structural damage to metabolic compensation and ultimately to multi-pathway stress. Previous studies have reported persistent activation of TLRs and NLRP3 in diabetic kidney disease (DKD), IgAN, and lupus nephritis, while abnormalities or mutations in TRPC6 calcium channels are common in focal segmental glomerulosclerosis and DKD. There are common molecular mechanisms underlying podocyte injury across kidney diseases. The latter stages of the trajectory were marked by metabolic dysregulation, inflammatory activation, cytoskeletal remodeling, organelle imbalance, and signaling pathway disturbances, consistent with responses previously observed under disturbed microenvironments (3). At this stage, podocytes exhibited depleted compensatory capacity, suggesting progression from reversible injury to functional imbalance or irreversible damage. Taken together, these results suggest that podocyte injury exhibits similar adaptive responses across different pathological states. These distinct triggers (e.g., HSPG2, ECM, and SPP1) may eventually converge through their respective pathways, leading to cytoskeletal rearrangement in podocytes and triggering a common downstream stress response. This process may represent an important adaptive response of podocytes to injury or stress, resulting in changes in cell morphology and function.

Based on these results, we raise several critical scientific questions for further investigation. First, it remains unclear whether podocytes activate similar molecular pathways in response to different types of injury. We hypothesize that podocytes exhibit shared adaptive responses, which may help identify therapeutic targets applicable across various kidney diseases. Additionally, in IgAN, the HSPG2-DAG1 axis may mediate mesangial cell–induced podocyte injury, and its mechanism and therapeutic potential require further investigation. In IMN, identifying key ECM components and their regulatory mechanisms could inform new strategies to protect podocytes. In AKI, *SPP1* is markedly upregulated in both the glomerulus and tubules and may serve as a non-invasive urinary biomarker, though its diagnostic and prognostic value remains to be validated.

We acknowledge several limitations of our study. First, due to the small sample size and batch effects, only three kidney diseases were included. This limits the generalizability of our findings and the identification of shared molecular pathways. Additionally, the analysis was based on a relatively small number of podocytes, which were subdivided into four subpopulations across four conditions. The limited number of cells per subpopulation may impose constraints on the statistical power and accuracy of the subpopulation analysis. Second, our analysis relied primarily on preliminary bioinformatics approaches and lacked comprehensive functional validation of key molecular mechanisms and potential therapeutic targets. Future studies should increase sample sizes, broaden the range of kidney diseases examined, and integrate multi-omics approaches with *in vivo* and *in vitro* functional experiments. These efforts will better elucidate molecular mechanisms and validate critical targets, providing a firmer theoretical foundation for clinical applications.

## 5 Conclusion

In summary, our study demonstrates that various types of podocyte injury follow a similar differentiation trajectory, offering new insights into the mechanisms underlying podocyte responses to injury.

## Data Availability

The original contributions presented in the study are included in the article/**Supplementary Material**. Further inquiries can be directed to the corresponding authors.

## References

[1] Shankland SJ . The podocyte’s response to injury: role in proteinuria and glomerulosclerosis. Kidney Int. (2006) 69:2131–47. doi: 10.1038/sj.ki.5000410, PMID: 16688120

[2] Schell C Huber TB . The evolving complexity of the podocyte cytoskeleton. J Am Soc Nephrology: JASN. (2017) 28:3166–74. doi: 10.1681/ASN.2017020143, PMID: 28864466 PMC5661293

[3] Bertram JF Cullen-McEwen LA Andrade-Oliveira V Câmara NOS . The intelligent podocyte: sensing and responding to a complex microenvironment. Nat Rev Nephrology. (2025) 21:503–16. doi: 10.1038/s41581-025-00965-y, PMID: 40341763

[4] Lal MA Young KW Andag U . Targeting the podocyte to treat glomerular kidney disease. Drug Discov Today. (2015) 20:1228–34. doi: 10.1016/j.drudis.2015.06.003, PMID: 26096184

[5] Asanuma K . The role of podocyte injury in chronic kidney disease. Nihon Rinsho Men’eki Gakkai kaishi = Japanese J Clin Immunol. (2015) 38:26–36. doi: 10.2177/jsci.38.26, PMID: 25765686

[6] Loreth D Sachs W Meyer-Schwesinger C . The life of a kidney podocyte. Acta physiologica (Oxford England). (2025) 241:e70081. doi: 10.1111/apha.70081, PMID: 40698593 PMC12284917

[7] Greka A Mundel P . Cell biology and pathology of podocytes. Annu Rev Physiol. (2012) 74:299–323. doi: 10.1146/annurev-physiol-020911-153238, PMID: 22054238 PMC3600372

[8] Alquraishi M Chahed S Alani D Puckett DL Dowker PD Hubbard K . Podocyte specific deletion of PKM2 ameliorates LPS-induced podocyte injury through beta-catenin. Cell communication signaling: CCS. (2022) 20:76. doi: 10.1186/s12964-022-00884-6, PMID: 35637461 PMC9150347

[9] Meliambro K He JC Campbell KN . Podocyte-targeted therapies - progress and future directions. Nat Rev Nephrology. (2024) 20:643–58. doi: 10.1038/s41581-024-00843-z, PMID: 38724717

[10] Leeuwis JW Nguyen TQ Dendooven A Kok RJ Goldschmeding R . Targeting podocyte-associated diseases. Advanced Drug delivery Rev. (2010) 62:1325–36. doi: 10.1016/j.addr.2010.08.012, PMID: 20828590

[11] Lu CC Wang GH Lu J Chen PP Zhang Y Hu ZB . Role of podocyte injury in glomerulosclerosis. Adv Exp Med Biol. (2019) 1165:195–232. doi: 10.1007/978-981-13-8871-2_10, PMID: 31399967 PMC7120923

[12] Bejoy J Qian ES Woodard LE . Tissue culture models of AKI: from tubule cells to human kidney organoids. J Am Soc Nephrology: JASN. (2022) 33:487–501. doi: 10.1681/ASN.2021050693, PMID: 35031569 PMC8975068

[13] Rayego-Mateos S Marquez-Expósito L Rodrigues-Diez R Sanz AB Guiteras R Doladé N . Molecular mechanisms of kidney injury and repair. Int J Mol Sci. (2022) 23:1542. doi: 10.3390/ijms23031542, PMID: 35163470 PMC8835923

[14] Takahashi M Yamamoto S Yamamoto S Okubo A Nakagawa Y Kuwahara K . ATP dynamics as a predictor of future podocyte structure and function after acute ischemic kidney injury in female mice. Nat Commun. (2024) 15:9977. doi: 10.1038/s41467-024-54222-0, PMID: 39578451 PMC11584722

[15] Schult L Halbgebauer R Karasu E Huber-Lang M . Glomerular injury after trauma, burn, and sepsis. J nephrology. (2023) 36:2417–29. doi: 10.1007/s40620-023-01718-5, PMID: 37542608 PMC10703988

[16] Chen Y Lin L Rao S Tao X Cui J Wan J . Complement C3 mediates podocyte injury through TLR4/NFKB-P65 signaling during ischemia-reperfusion acute kidney injury and post-injury fibrosis. Eur J Med Res. (2023) 28:135. doi: 10.1186/s40001-023-01054-1, PMID: 36973754 PMC10041728

[17] Hu X Zhou W Wu S Wang R Luan Z Geng X . Tacrolimus alleviates LPS-induced AKI by inhibiting TLR4/MyD88/NF-κB signalling in mice. J Cell Mol Med. (2022) 26:507–14. doi: 10.1111/jcmm.17108, PMID: 34889045 PMC8743665

[18] Gong Q Lai T Liang L Jiang Y Liu F . Targeted inhibition of CX3CL1 limits podocytes ferroptosis to ameliorate cisplatin-induced acute kidney injury. Mol Med (Cambridge Mass). (2023) 29:140. doi: 10.1186/s10020-023-00733-3, PMID: 37875838 PMC10594885

[19] Chen Y Lin L Tao X Song Y Cui J Wan J . The role of podocyte damage in the etiology of ischemia-reperfusion acute kidney injury and post-injury fibrosis. BMC nephrology. (2019) 20:106. doi: 10.1186/s12882-019-1298-x, PMID: 30922260 PMC6438002

[20] Stuart T Satija R . Integrative single-cell analysis. Nat Rev Genet. (2019) 20:257–72. doi: 10.1038/s41576-019-0093-7, PMID: 30696980

[21] Tang R Lin W Shen C Hu X Yu L Meng T . Single-cell transcriptomics uncover hub genes and cell-cell crosstalk in patients with hypertensive nephropathy. Int Immunopharmacol. (2023) 125:111104. doi: 10.1016/j.intimp.2023.111104, PMID: 37897949

[22] Tang R Meng T Lin W Shen C Ooi JD Eggenhuizen PJ . A partial picture of the single-cell transcriptomics of human igA nephropathy. Front Immunol. (2021) 12:645988. doi: 10.3389/fimmu.2021.645988, PMID: 33936064 PMC8085501

[23] Xu J Shen C Lin W Meng T Ooi JD Eggenhuizen PJ . Single-cell profiling reveals transcriptional signatures and cell-cell crosstalk in anti-PLA2R positive idiopathic membranous nephropathy patients. Front Immunol. (2021) 12:683330. doi: 10.3389/fimmu.2021.683330, PMID: 34135910 PMC8202011

[24] Tang R Jin P Shen C Lin W Yu L Hu X . Single-cell RNA sequencing reveals the transcriptomic landscape of kidneys in patients with ischemic acute kidney injury. Chin Med J. (2023) 136:1177–87. doi: 10.1097/CM9.0000000000002679, PMID: 37083129 PMC10278705

[25] Slovin S Carissimo A Panariello F Grimaldi A Bouché V Gambardella G . Single-cell RNA sequencing analysis: A step-by-step overview. Methods Mol Biol (Clifton NJ). (2021) 2284:343–65. doi: 10.1007/978-1-0716-1307-8_19, PMID: 33835452

[26] McGinnis CS Murrow LM Gartner ZJ . DoubletFinder: doublet detection in single-cell RNA sequencing data using artificial nearest neighbors. Cell systems. (2019) 8:329–37.e4. doi: 10.1016/j.cels.2019.03.003, PMID: 30954475 PMC6853612

[27] Korsunsky I Millard N Fan J Slowikowski K Zhang F Wei K . Fast, sensitive and accurate integration of single-cell data with Harmony. Nat Methods. (2019) 16:1289–96. doi: 10.1038/s41592-019-0619-0, PMID: 31740819 PMC6884693

[28] Lake BB Menon R Winfree S Hu Q Melo Ferreira R Kalhor K . An atlas of healthy and injured cell states and niches in the human kidney. Nature. (2023) 619:585–94. doi: 10.1038/s41586-023-05769-3, PMID: 37468583 PMC10356613

[29] Gu X Jiang K Chen R Chen Z Wu X Xiang H . Identification of common stria vascularis cellular alteration in sensorineural hearing loss based on ScRNA-seq. BMC Genomics. (2024) 25:213. doi: 10.1186/s12864-024-10122-7, PMID: 38413848 PMC10897997

[30] Wu T Hu E Xu S Chen M Guo P Dai Z . clusterProfiler 4.0: A universal enrichment tool for interpreting omics data. Innovation (Cambridge (Mass)). (2021) 2:100141. doi: 10.1016/j.xinn.2021.100141, PMID: 34557778 PMC8454663

[31] Jin S Guerrero-Juarez CF Zhang L Chang I Ramos R Kuan CH . Inference and analysis of cell-cell communication using CellChat. Nat Commun. (2021) 12:1088. doi: 10.1038/s41467-021-21246-9, PMID: 33597522 PMC7889871

[32] Liu WB Huang GR Liu BL Hu HK Geng J Rui HL . Single cell landscape of parietal epithelial cells in healthy and diseased states. Kidney Int. (2023) 104:108–23. doi: 10.1016/j.kint.2023.03.036, PMID: 37100348

[33] Subramanian A Tamayo P Mootha VK Mukherjee S Ebert BL Gillette MA . Gene set enrichment analysis: a knowledge-based approach for interpreting genome-wide expression profiles. Proc Natl Acad Sci United States America. (2005) 102:15545–50. doi: 10.1073/pnas.0506580102, PMID: 16199517 PMC1239896

[34] Andrews GK . Regulation of metallothionein gene expression by oxidative stress and metal ions. Biochem Pharmacol. (2000) 59:95–104. doi: 10.1016/S0006-2952(99)00301-9, PMID: 10605938

[35] Dabravolski SA . Mitochondria-derived peptides in healthy ageing and therapy of age-related diseases. Adv Protein Chem Struct Biol. (2023) 136:197–215. doi: 10.1016/bs.apcsb.2023.02.015, PMID: 37437978

[36] Mirzaei MR Kazemi Arababadi M Asadi MH Mowla SJ . Altered expression of high molecular weight heat shock proteins after OCT4B1 suppression in human tumor cell lines. Cell J. (2016) 17:608–16. doi: 10.22074/cellj.2016.3832, PMID: 26862520 PMC4746411

[37] Peng R Lin H Zhu H Zhang Y Bao T Li W . Involvement of IGF1 in endoplasmic reticulum stress contributes to cataract formation through regulating Nrf2/NF-κB signaling. Funct Integr Genomics. (2023) 23:220. doi: 10.1007/s10142-023-01152-7, PMID: 37394478

[38] Tang Z Zhang Z Zhao J Zhang F Zhang Y Wen Y . Integrated analysis of multiple programmed cell death-related prognostic genes and functional validation of apoptosis-related genes in osteosarcoma. Int J Biol macromolecules. (2025) 307:142113. doi: 10.1016/j.ijbiomac.2025.142113, PMID: 40089239

[39] Zhang T Yang S Ge Y Wan X Zhu Y Li J . Polystyrene nanoplastics induce lung injury via activating oxidative stress: molecular insights from bioinformatics analysis. Nanomaterials (Basel Switzerland). (2022) 12:3507. doi: 10.3390/nano12193507, PMID: 36234635 PMC9565894

[40] Fernando N Wooff Y Aggio-Bruce R Chu-Tan JA Jiao H Dietrich C . Photoreceptor survival is regulated by GSTO1–1 in the degenerating retina. Invest Ophthalmol Visual science. (2018) 59:4362–74. doi: 10.1167/iovs.18-24627, PMID: 30193308

[41] Gulati GS Sikandar SS Wesche DJ Manjunath A Bharadwaj A Berger MJ . Single-cell transcriptional diversity is a hallmark of developmental potential. Sci (New York NY). (2020) 367:405–11. doi: 10.1126/science.aax0249, PMID: 31974247 PMC7694873

[42] Cao J Spielmann M Qiu X Huang X Ibrahim DM Hill AJ . The single-cell transcriptional landscape of mammalian organogenesis. Nature. (2019) 566:496–502. doi: 10.1038/s41586-019-0969-x, PMID: 30787437 PMC6434952

[43] Qiu X Mao Q Tang Y Wang L Chawla R Pliner HA . Reversed graph embedding resolves complex single-cell trajectories. Nat Methods. (2017) 14:979–82. doi: 10.1038/nmeth.4402, PMID: 28825705 PMC5764547

[44] Saelens W Cannoodt R Todorov H Saeys Y . A comparison of single-cell trajectory inference methods. Nat Biotechnol. (2019) 37:547–54. doi: 10.1038/s41587-019-0071-9, PMID: 30936559

[45] Zhang F Li X Tian W . Unsupervised inference of developmental directions for single cells using VECTOR. Cell Rep. (2020) 32:108069. doi: 10.1016/j.celrep.2020.108069, PMID: 32846127

[46] Lu Y Ye Y Bao W Yang Q Wang J Liu Z . Genome-wide identification of genes essential for podocyte cytoskeletons based on single-cell RNA sequencing. Kidney Int. (2017) 92:1119–29. doi: 10.1016/j.kint.2017.04.022, PMID: 28709640

[47] Chen J Boyle S Zhao M Su W Takahashi K Davis L . Differential expression of the intermediate filament protein nestin during renal development and its localization in adult podocytes. J Am Soc Nephrology: JASN. (2006) 17:1283–91. doi: 10.1681/ASN.2005101032, PMID: 16571784

[48] Csurgyók R Sütő G Wittmann I Vas T . Expression of wilms’ Tumor 1 antigen, vimentin, and corticotropin-releasing factor in the human kidney with focal segmental glomerulosclerosis and effect of oxidative stress on these markers in HEK 293 cells. Kidney Blood Pressure Res. (2023) 48:56–65. doi: 10.1159/000528727, PMID: 36529126 PMC9909720

[49] Pierchala BA Muñoz MR Tsui CC . Proteomic analysis of the slit diaphragm complex: CLIC5 is a protein critical for podocyte morphology and function. Kidney Int. (2010) 78:868–82. doi: 10.1038/ki.2010.212, PMID: 20664558 PMC5538030

[50] Mason WJ Vasilopoulou E . The pathophysiological role of thymosin β4 in the kidney glomerulus. Int J Mol Sci. (2023) 24:7684. doi: 10.3390/ijms24097684, PMID: 37175390 PMC10177875

[51] Franceschini N North KE Kopp JB McKenzie L Winkler C . NPHS2 gene, nephrotic syndrome and focal segmental glomerulosclerosis: a HuGE review. Genet Med. (2006) 8:63–75. doi: 10.1097/01.gim.0000200947.09626.1c, PMID: 16481888

[52] Bunnell TM Burbach BJ Shimizu Y Ervasti JM . β-Actin specifically controls cell growth, migration, and the G-actin pool. Mol Biol Cell. (2011) 22:4047–58. doi: 10.1091/mbc.e11-06-0582, PMID: 21900491 PMC3204067

[53] Kraja AT Liu C Fetterman JL Graff M Have CT Gu C . Associations of mitochondrial and nuclear mitochondrial variants and genes with seven metabolic traits. Am J Hum Genet. (2019) 104:112–38. doi: 10.1016/j.ajhg.2018.12.001, PMID: 30595373 PMC6323610

[54] Tavleeva MM Belykh ES Rybak AV Rasova EE Chernykh AA Ismailov ZB . Effects of antioxidant gene overexpression on stress resistance and Malignization *in vitro* and *in vivo*: A review. Antioxidants (Basel Switzerland). (2022) 11:2316. doi: 10.3390/antiox11122316, PMID: 36552527 PMC9774954

[55] Ruttkay-Nedecky B Nejdl L Gumulec J Zitka O Masarik M Eckschlager T . The role of metallothionein in oxidative stress. Int J Mol Sci. (2013) 14:6044–66. doi: 10.3390/ijms14036044, PMID: 23502468 PMC3634463

[56] Miao J Fan Q Cui Q Zhang H Chen L Wang S . Newly identified cytoskeletal components are associated with dynamic changes of podocyte foot processes. Nephrology dialysis Transplant. (2009) 24:3297–305. doi: 10.1093/ndt/gfp338, PMID: 19617259

[57] Vad OB Paludan-Müller C Ahlberg G Kalstø SM Ghouse J Andreasen L . Loss-of-function variants in cytoskeletal genes are associated with early-onset atrial fibrillation. J Clin Med. (2020) 9:372. doi: 10.3390/jcm9020372, PMID: 32013268 PMC7074234

[58] Baudier J Jenkins ZA Robertson SP . The filamin-B-refilin axis - spatiotemporal regulators of the actin-cytoskeleton in development and disease. J Cell Sci. (2018) 131:jcs213959. doi: 10.1242/jcs.213959, PMID: 29654161

[59] Herman MA Birnbaum MJ . Molecular aspects of fructose metabolism and metabolic disease. Cell Metab. (2021) 33:2329–54. doi: 10.1016/j.cmet.2021.09.010, PMID: 34619074 PMC8665132

[60] Nemkov T Key A Stephenson D Earley EJ Keele GR Hay A . Genetic regulation of carnitine metabolism controls lipid damage repair and aging RBC hemolysis *in vivo* and *in vitro*. Blood. (2024) 143:2517–33. doi: 10.1182/blood.2024023983, PMID: 38513237 PMC11208298

[61] Yuan Y Wang C Zhuang X Lin S Luo M Deng W . PIM1 promotes hepatic conversion by suppressing reprogramming-induced ferroptosis and cell cycle arrest. Nat Commun. (2022) 13:5237. doi: 10.1038/s41467-022-32976-9, PMID: 36068222 PMC9448736

[62] Roberts MA Deol KK Mathiowetz AJ Lange M Leto DE Stevenson J . Parallel CRISPR-Cas9 screens identify mechanisms of PLIN2 and lipid droplet regulation. Dev Cell. (2023) 58:1782–800.e10. doi: 10.1016/j.devcel.2023.07.001, PMID: 37494933 PMC10530302

[63] Wu C Dai C Li X Sun M Chu H Xuan Q . AKR1C3-dependent lipid droplet formation confers hepatocellular carcinoma cell adaptability to targeted therapy. Theranostics. (2022) 12:7681–98. doi: 10.7150/thno.74974, PMID: 36451864 PMC9706585

[64] Aass KR Kastnes MH Standal T . Molecular interactions and functions of IL-32. J leukocyte Biol. (2021) 109:143–59. doi: 10.1002/JLB.3MR0620-550R, PMID: 32869391

[65] Singh V Kaur R Kumari P Pasricha C Singh R . ICAM-1 and VCAM-1: Gatekeepers in various inflammatory and cardiovascular disorders. Clinica chimica Acta. (2023) 548:117487. doi: 10.1016/j.cca.2023.117487, PMID: 37442359

[66] Yi F Frazzette N Cruz AC Klebanoff CA Siegel RM . Beyond cell death: new functions for TNF family cytokines in autoimmunity and tumor immunotherapy. Trends Mol Med. (2018) 24:642–53. doi: 10.1016/j.molmed.2018.05.004, PMID: 29880309 PMC7466867

[67] Ramirez F Tanaka S Bou-Gharios G . Transcriptional regulation of the human alpha2(I) collagen gene (COL1A2), an informative model system to study fibrotic diseases. Matrix biology: J Int Soc Matrix Biol. (2006) 25:365–72. doi: 10.1016/j.matbio.2006.05.002, PMID: 16815696

[68] Song J Chen Y Chen Y Qiu M Xiang W Ke B . DKK3 promotes renal fibrosis by increasing MFF-mediated mitochondrial dysfunction in Wnt/β-catenin pathway-dependent manner. Renal failure. (2024) 46:2343817. doi: 10.1080/0886022X.2024.2343817, PMID: 38682264 PMC11060011

[69] Ding H Xu Z Lu Y Yuan Q Li J Sun Q . Kidney fibrosis molecular mechanisms Spp1 influences fibroblast activity through transforming growth factor beta smad signaling. iScience. (2024) 27:109839. doi: 10.1016/j.isci.2024.109839, PMID: 39323737 PMC11422156

[70] Johnson BB Cosson MV Tsansizi LI Holmes TL Gilmore T Hampton K . Perlecan (HSPG2) promotes structural, contractile, and metabolic development of human cardiomyocytes. Cell Rep. (2024) 43:113668. doi: 10.1016/j.celrep.2023.113668, PMID: 38198277

[71] Goldberg S Harvey SJ Cunningham J Tryggvason K Miner JH . Glomerular filtration is normal in the absence of both agrin and perlecan-heparan sulfate from the glomerular basement membrane. Nephrology dialysis Transplant. (2009) 24:2044–51. doi: 10.1093/ndt/gfn758, PMID: 19144998 PMC2721481

[72] Regele HM Fillipovic E Langer B Poczewki H Kraxberger I Bittner RE . Glomerular expression of dystroglycans is reduced in minimal change nephrosis but not in focal segmental glomerulosclerosis. J Am Soc Nephrology: JASN. (2000) 11:403–12. doi: 10.1681/ASN.V113403, PMID: 10703664

[73] Kuivaniemi H Tromp G . Type III collagen (COL3A1): Gene and protein structure, tissue distribution, and associated diseases. Gene. (2019) 707:151–71. doi: 10.1016/j.gene.2019.05.003, PMID: 31075413 PMC6579750

[74] Heidet L Arrondel C Forestier L Cohen-Solal L Mollet G Gutierrez B . Structure of the human type IV collagen gene COL4A3 and mutations in autosomal Alport syndrome. J Am Soc Nephrology: JASN. (2001) 12:97–106. doi: 10.1681/ASN.V12197, PMID: 11134255

[75] Madne TH Dockrell MEC . TGFβ1-mediated PI3K/Akt and p38 MAP kinase dependent alternative splicing of fibronectin extra domain A in human podocyte culture. Cell Mol Biol (Noisy-le-Grand France). (2018) 64:127–35., PMID: 29729706

[76] Byron A Randles MJ Humphries JD Mironov A Hamidi H Harris S . Glomerular cell cross-talk influences composition and assembly of extracellular matrix. J Am Soc Nephrology: JASN. (2014) 25:953–66. doi: 10.1681/ASN.2013070795, PMID: 24436469 PMC4005312

[77] Lennon R Randles MJ Humphries MJ . The importance of podocyte adhesion for a healthy glomerulus. Front endocrinology. (2014) 5:160. doi: 10.3389/fendo.2014.00160, PMID: 25352829 PMC4196579

[78] Grahammer F Schell C Huber TB . The podocyte slit diaphragm–from a thin grey line to a complex signalling hub. Nat Rev Nephrology. (2013) 9:587–98. doi: 10.1038/nrneph.2013.169, PMID: 23999399

[79] Bock F Li S Pozzi A Zent R . Integrins in the kidney - beyond the matrix. Nat Rev Nephrology. (2025) 21:157–74. doi: 10.1038/s41581-024-00906-1, PMID: 39643697 PMC12640780

[80] Li L Fu H Liu Y . The fibrogenic niche in kidney fibrosis: components and mechanisms. Nat Rev Nephrology. (2022) 18:545–57. doi: 10.1038/s41581-022-00590-z, PMID: 35788561

[81] Sun Z Guo SS Fässler R . Integrin-mediated mechanotransduction. J Cell Biol. (2016) 215:445–56. doi: 10.1083/jcb.201609037, PMID: 27872252 PMC5119943

[82] Sharma-Walia N Naranatt PP Krishnan HH Zeng L Chandran B . Kaposi’s sarcoma-associated herpesvirus/human herpesvirus 8 envelope glycoprotein gB induces the integrin-dependent focal adhesion kinase-Src-phosphatidylinositol 3-kinase-rho GTPase signal pathways and cytoskeletal rearrangements. J virology. (2004) 78:4207–23. doi: 10.1128/JVI.78.8.4207-4223.2004, PMID: 15047836 PMC374261

[83] Christaki F Ghasemi A Pantham D Abouali R Provera A Vecchio C . Role of balanced involvement of the ICOS/ICOSL/osteopontin network in cutaneous wound healing. Int J Mol Sci. (2024) 25:12390. doi: 10.3390/ijms252212390, PMID: 39596455 PMC11594701

[84] Xu Q Li ZL Zhang YL Wu M Shen AR Tang TT . Kidney hepcidin protects the collecting duct against ferroptosis in ischemia/reperfusion-induced acute kidney injury. Kidney Int. (2025) 108:394–410. doi: 10.1016/j.kint.2025.05.030, PMID: 40581167

[85] Fogo AB Harris RC . Crosstalk between glomeruli and tubules. Nat Rev Nephrology. (2025) 21:189–99. doi: 10.1038/s41581-024-00907-0, PMID: 39643696

[86] Zhao Y Huang Z Gao L Ma H Chang R . Osteopontin/SPP1: a potential mediator between immune cells and vascular calcification. Front Immunol. (2024) 15:1395596. doi: 10.3389/fimmu.2024.1395596, PMID: 38919629 PMC11196619

[87] Tian X Ishibe S . Targeting the podocyte cytoskeleton: from pathogenesis to therapy in proteinuric kidney disease. Nephrology dialysis Transplant. (2016) 31:1577–83. doi: 10.1093/ndt/gfw021, PMID: 26968197 PMC5039341

[88] Yu G Wang X Luo J Su X Tao H Wen Z . Role of SPP1 in acute kidney injury induced by renal ischemia-reperfusion in rats. Nan fang yi ke da xue xue bao = J South Med University. (2023) 43:1947–54. doi: 10.12122/j.issn.1673-4254.2023.11.16, PMID: 38081614 PMC10713472

[89] Pang S Zhou R Liu Z Xie B Liu F Feng B . SPP1 as a biomarker for idiopathic membranous nephropathy progression and its regulatory role in inflammation and fibrosis. Front Immunol. (2025) 16:1671891. doi: 10.3389/fimmu.2025.1671891, PMID: 41080598 PMC12510867

[90] Fu Z Geng X Liu C Shen W Dong Z Sun G . Identification of common and specific fibrosis-related genes in three common chronic kidney diseases. Renal failure. (2024) 46:2295431. doi: 10.1080/0886022X.2023.2295431, PMID: 38174742 PMC10769532

[91] Yang X Liu Z Zhou J Guo J Han T Liu Y . SPP1 promotes the polarization of M2 macrophages through the Jak2/Stat3 signaling pathway and accelerates the progression of idiopathic pulmonary fibrosis. . Int J Mol Med. (2024) 54:89. doi: 10.3892/ijmm.2024.5413, PMID: 39129313 PMC11335352

[92] Song Z Chen W Athavale D Ge X Desert R Das S . Osteopontin takes center stage in chronic liver disease. Hepatol (Baltimore Md). (2021) 73:1594–608. doi: 10.1002/hep.31582, PMID: 32986864 PMC8106357

[93] Avihingsanon Y Benjachat T Tassanarong A Sodsai P Kittikovit V Hirankarn N . Decreased renal expression of vascular endothelial growth factor in lupus nephritis is associated with worse prognosis. Kidney Int. (2009) 75:1340–8. doi: 10.1038/ki.2009.75, PMID: 19295501

