# Supplementary material for: Single-cell analysis reveals shared adaptive responses across different types of podocyte injury
Source: Front Immunol. 2025 Dec 16;16:1698284. doi: 10.3389/fimmu.2025.1698284 (PMC12747916; doi:10.3389/fimmu.2025.1698284)
Supplement: Supplementary file 1 [file Table1.docx]

Supplementary Material

# Supplementary Figures


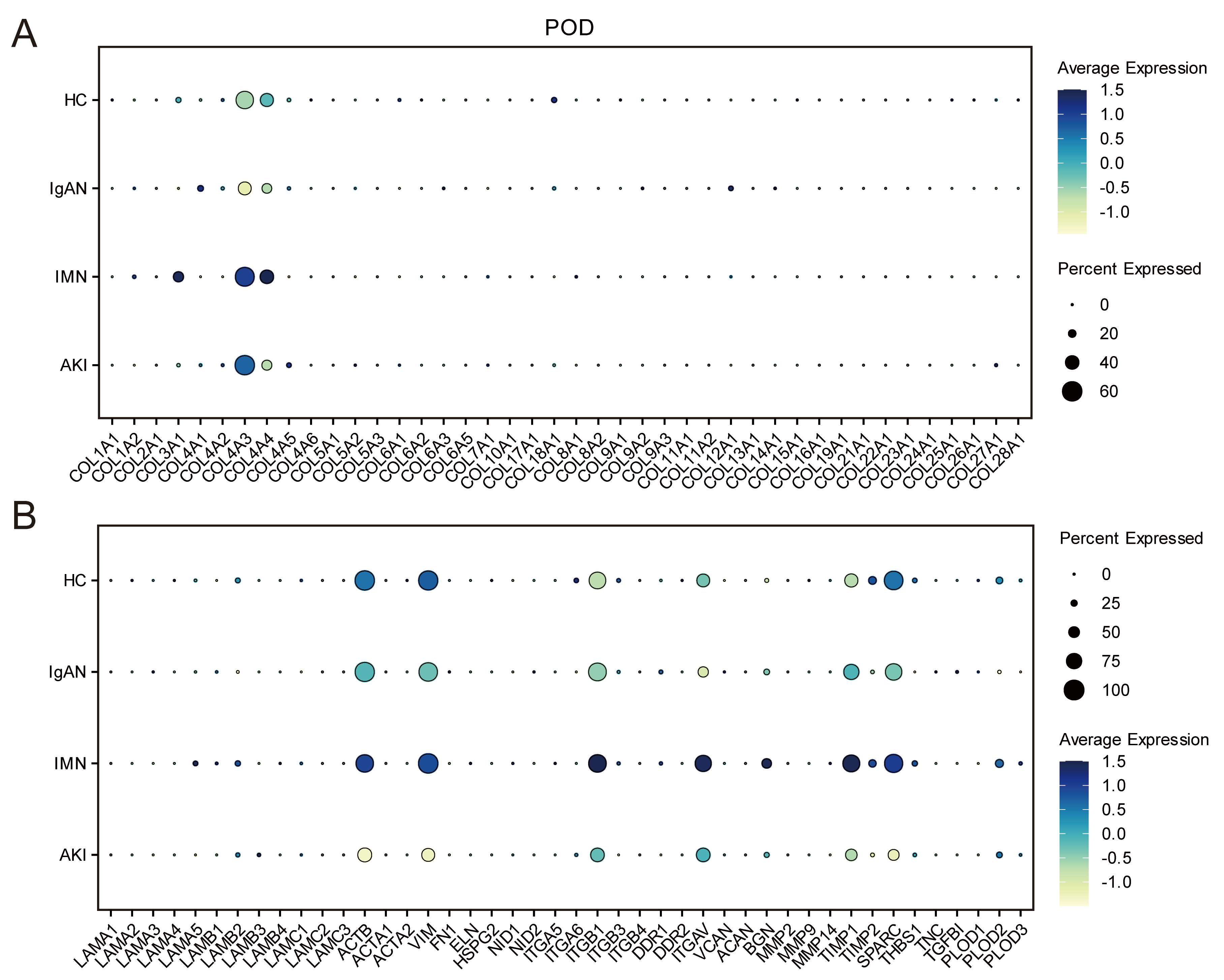


Supplementary Figure S1. Expression of collagen and extracellular matrix-related genes in podocytes. (A) Dot plot showing the expression of collagen genes in podocytes across groups. (B) Dot plot showing the expression of extracellular matrix-related genes in podocytes across groups.


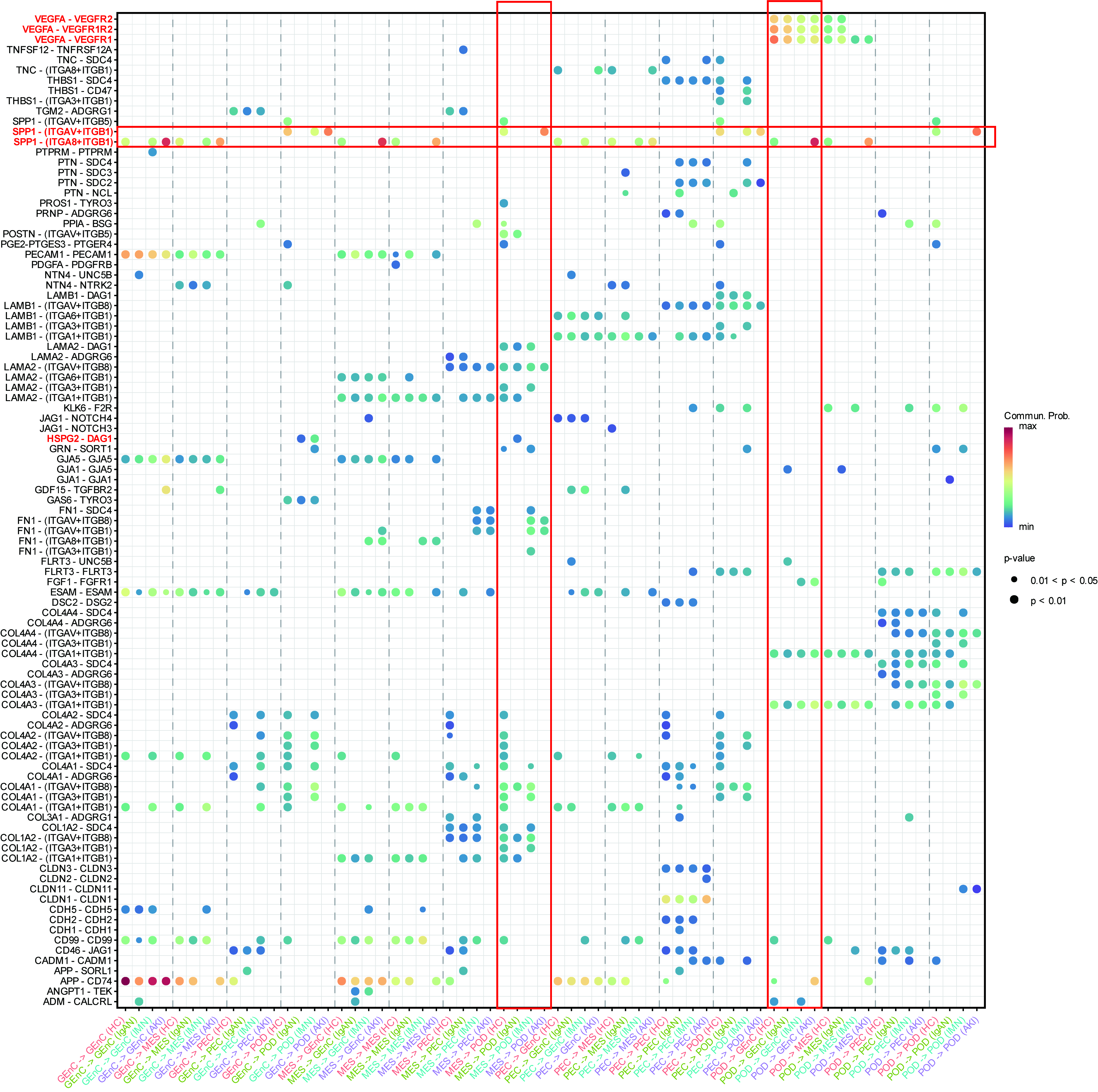


Supplementary Figure S2. CellChat analysis of interactions among glomerular endothelial cells, mesangial cells, parietal epithelial cells, and podocytes across the four groups: HC, IgAN, IMN, and AKI. The dot plot compares significant ligand–receptor pairs across the four groups. Dot color indicates communication probabilities, and dot size represents the calculated *P*-value. Blank areas correspond to a communication probability of zero. *P*-values were calculated using a one-sided permutation test. Red boxes and labels highlight the main ligand–receptor pairs.


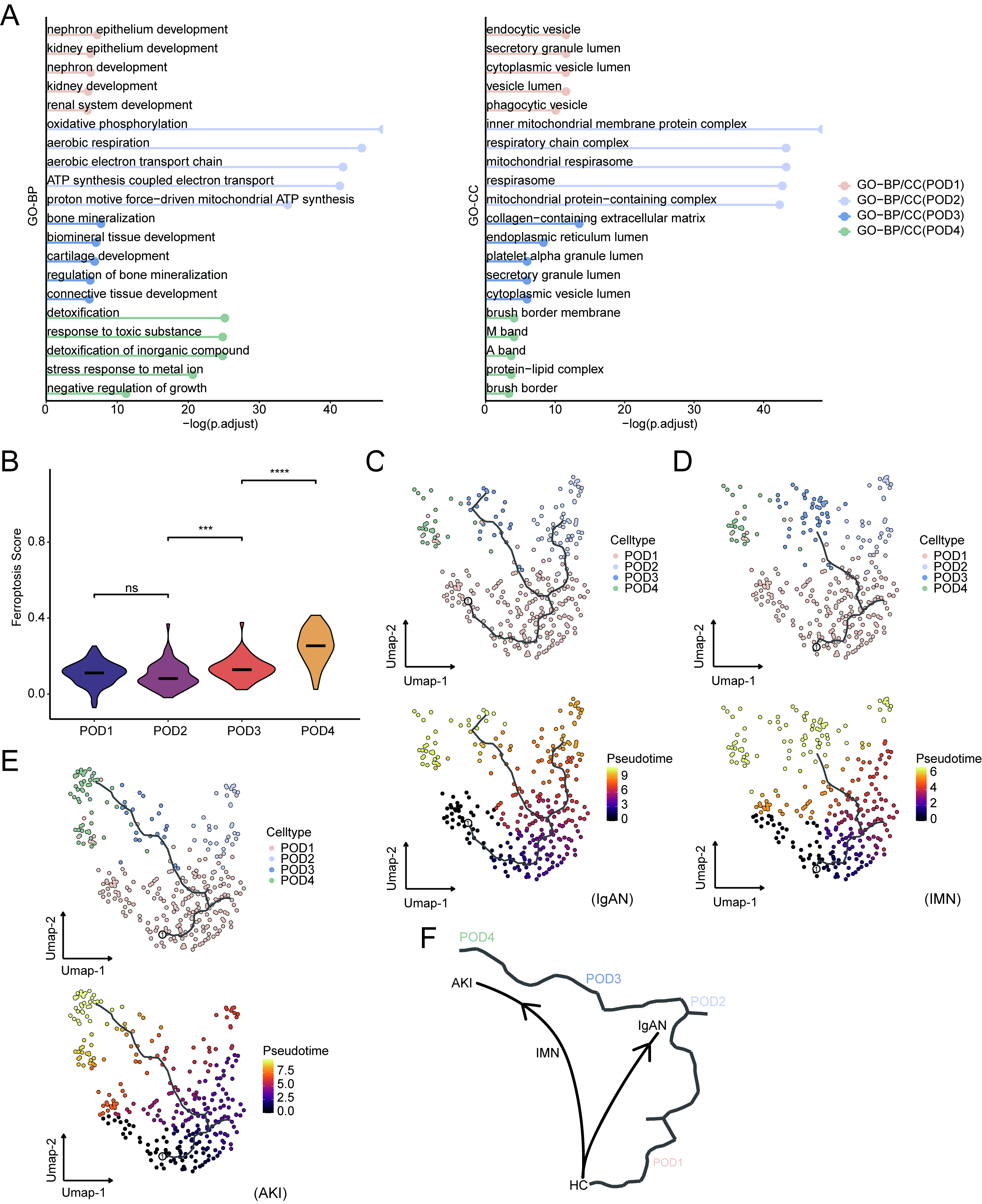


Supplementary Figure S3. Characterization of POD subpopulations and their differentiation trajectories across disease states. (A) Bar plots showing −log10 (adjusted *P*-values) of selected GO pathway enrichments in each subpopulation. (B) Violin plots displaying ferroptosis-related gene module scores across POD subpopulations. (C–E) Monocle 3 illustrates the trajectories of podocytes from HC to each disease state (IgAN, IMN, and AKI), respectively, with cells colored by subpopulation or pseudotime. (F) A schematic illustration of podocyte trajectories across various disease states, showing their primary differentiation directions. ****P* < 0.001, *****P* < 0.0001.


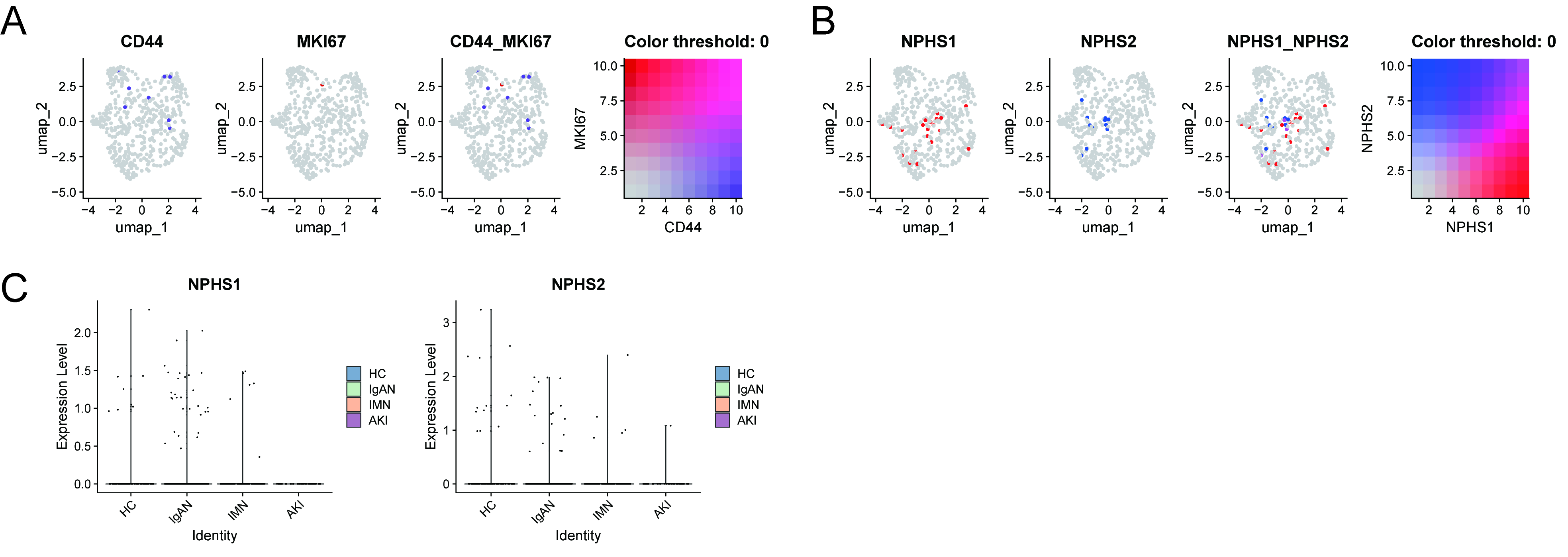


Supplementary Figure S4. (A) UMAP plot of CD44 and MKI67 expression in PECs as a dual marker. (B) UMAP plot of NPHS1 and NPHS2 expression in PECs as a dual marker. (C) Violin plot showing the expression of NPHS1 and NPHS2 in PECs.

# Supplementary Tables

**Supplementary Table S1.** **Clinical information of patients.**

|  | **Age** | **Gender** | **Scr (mg/dL)** | **eGFR(mL/min/1.73 m**^2^**)** | **Proteinuria (g﻿/24h)** | **Disease** |
| --- | --- | --- | --- | --- | --- | --- |
| GSM5225906  GSM5225907  GSM5222734  GSM5289542  GSM5222730  GSM5222731  GSM5222732  GSM5222733  GSM5225900  GSM5225901  GSM5225902  GSM5225903  GSM5225904  GSM5225905  GSM5289544  GSM5289545 | 47  50  41  48  26  50  39  20  64  61  47  65  52  34  45  53 | Male  Male  Male  Male  Male  Female  Female  Female  Male  Male  Female  Male  Male  Male  Male  Male | 0.68  0.94  0.94  0.96  2.59  0.94  0.70  0.76  1.17  0.97  0.63  1.14  1.09  1.46  3.28  2.84 | 113.81  94.16  100.47  93.09  32.72  70.84  109.65  114.09  65.75  84.32  107  66.93  77.58  61.91  NA  NA | none  none  none  none  2.57  0.56  1.42  0.27  10.75  3.54  1.18  8.02  2.34  11.35  NA  NA | HC  HC  HC  HC  IgAN  IgAN  IgAN  IgAN  IMN  IMN  IMN  IMN  IMN  IMN  AKI  AKI |

Scr, serum creatinine; eGFR, estimated glomerular filtration rate; HC, Healthy Control; IgAN, IgA Nephropathy; IMN, Idiopathic Membranous Nephropathy; AKI, Acute Kidney Injury. “NA” indicates missing data; for two AKI patients, urinalysis showed normal or trace proteinuria. Note: For the patient samples in this study, some clinical information in the GEO database differs from that reported in the original publications; the original publication data were used as the primary reference.

**Supplementary Table S2. scRNA-seq datasets of human kidney tissue.**

| **GEO accession** | **Web address** | **Sample** |
| --- | --- | --- |
| GSE174219 | https://www.ncbi.nlm.nih.gov/geo/query/acc.cgi?acc=GSE174219 | GSM5289542 |
| GSE171314 | https://www.ncbi.nlm.nih.gov/geo/query/acc.cgi?acc=GSE171314 | GSM5222730  GSM5222731  GSM5222732  GSM5222733  GSM5222734 |
| GSE171458 | https://www.ncbi.nlm.nih.gov/geo/query/acc.cgi?acc=GSE171458 | GSM5225900  GSM5225901  GSM5225902  GSM5225903  GSM5225904  GSM5225905  GSM5225906  GSM5225907 |
| GSE174220 | https://www.ncbi.nlm.nih.gov/geo/query/acc.cgi?acc=GSE174220 | GSM5289544  GSM5289545 |

**Supplementary Table S3. Reference for human kidney cell type markers.**

| **POD** | **PEC** | **PT** | **TAL** | **DCT** | **PC** | **IC** |
| --- | --- | --- | --- | --- | --- | --- |
| PODXL  CLIC5  NPHS2  NPHS1  NTNG1  WT1  PTPRQ | ALDH1A2  RBFOX1  CFH  VCAM1  CLDN1 | SLC13A1  CUBN  LRP2 | UMOD  SLC12A1  CASR | TRPM6  LHX1  KLHL3  FGF13  CNNM2  SLC12A3 | AQP3  AQP2  GATA3 | CLNK  TMEM213  ATP6V1C2  ATP6V0D2 |

| **IMM** | **END** | **VSMC/P** | **CC** | **MES** | **GEnC** |
| --- | --- | --- | --- | --- | --- |
| PTPRC | EMCN  FLT1  MEIS2  PTPRB  PECAM1  CD34 | ITGA8  PDGFRB  NOTCH3 | MKI67  TOP2A | PIP5K1B  ROBO1  PIEZO2  DAAM2  PHTF2  GATA3  POSTN | EMCN  HECW2  PLAT  ITGA8  EHD3  KDR  SOST |

POD, Podocytes; PEC, Parietal epithelial cells; PT, Proximal tubule cells; TAL, Thick ascending limb cells; DCT, Distal convoluted tubule cells; PC, Principal cells; IC, Intercalated cells; IMM, Immune cells; END, Endothelial cells; VSMC/P, Vascular smooth muscle cells / Pericytes; CC, Cycling cells; MES, Mesangial cells; GEnC, Glomerular endothelial cells.

**Supplementary Table S4.** **Clinical information of patients for immunohistochemistry.**

| **Diagnosis** | **Age** | **Gender** |
| --- | --- | --- |
| IgAN  AKI  HC | 19  65  77 | male  male  female |

**Supplementary Table S5. Antibody information.**

| **Antibody** | **Source** | **Identifier** | **Dilution ratio in IHC** |
| --- | --- | --- | --- |
| Mouse anti-HSPG2  Mouse anti-SPP1 | Thermo Fisher Scientific  Thermo Fisher Scientific | 13-4400  MA5-17180 | 1:100  1:1000 |
